# RNase W, a conserved ribonuclease family with a novel active site

**DOI:** 10.1093/nar/gkae907

**Published:** 2024-10-24

**Authors:** Marlène Vayssières, Michael Jüttner, Karina Haas, Aurélie Ancelin, Anita Marchfelder, Nicolas Leulliot, Sébastien Ferreira-Cerca, Magali Blaud

**Affiliations:** Université Paris Cité, CNRS, CiTCoM, 4 avenue de l'Observatoire, F-75006 Paris, France; Regensburg Centre for Biochemistry, Biochemistry III—Institute for Biochemistry, Genetics and Microbiology, University of Regensburg, Universitätsstraße 31, 93053 Regensburg, Germany; Molecular Biology and Biotechnology of Prokaryotes, Ulm University, Albert-Einstein-Allee 11, 89069 Ulm, Germany; Université Paris Cité, CNRS, CiTCoM, 4 avenue de l'Observatoire, F-75006 Paris, France; Molecular Biology and Biotechnology of Prokaryotes, Ulm University, Albert-Einstein-Allee 11, 89069 Ulm, Germany; Université Paris Cité, CNRS, CiTCoM, 4 avenue de l'Observatoire, F-75006 Paris, France; Regensburg Centre for Biochemistry, Biochemistry III—Institute for Biochemistry, Genetics and Microbiology, University of Regensburg, Universitätsstraße 31, 93053 Regensburg, Germany; Laboratoire de Biologie Structurale de la Cellule (BIOC), UMR 7654—CNRS, École polytechnique, Institut Polytechnique de Paris, Route de Saclay, 91128 Palaiseau, France; Université Paris Cité, CNRS, CiTCoM, 4 avenue de l'Observatoire, F-75006 Paris, France

## Abstract

Ribosome biogenesis is a complex process requiring multiple precursor ribosomal RNA (rRNA) cleavage steps. In archaea, the full set of ribonucleases (RNases) involved in rRNA processing remains to be discovered. A previous study suggested that FAU-1, a conserved protein containing an RNase G/E-like protein domain fused to a domain of unknown function (DUF402), acts as an RNase in archaea. However, the molecular basis of this activity remained so far elusive. Here, we report two X-ray crystallographic structures of RNase G/E-like–DUF402 hybrid proteins from *Pyrococcus furiosus* and *Sulfolobus acidocaldarius*, at 2.1 and 2.0 Å, respectively. The structures highlight a structural homology with the 5′ RNA recognition domain of *Escherichia coli* RNase E but no homology with other known catalytic nuclease domains. Surprisingly, we demonstrate that the C-terminal domain of this hybrid protein, annotated as a putative diphosphatase domain, harbors the RNase activity. Our functional analysis also supports a model by which the RNase G/E-like domain acts as a regulatory subunit of the RNase activity. Finally, *in vivo* experiments in *Haloferax volcanii* suggest that this RNase participates in the maturation of pre-16S rRNA. Together, our study defines a new RNase family, which we termed the RNase W family, as the first archaea-specific contributor to archaeal ribosome biogenesis.

## Graphical Abstract

**Graphical Abstract:**
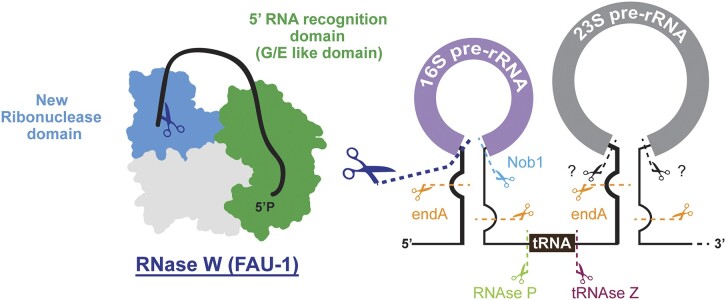

## Introduction

Ribosome biogenesis is a complex process that has been best characterized in bacteria and eukaryotes (1–6). However, due to the lack of conservation between the bacterial and eukaryotic ribosome biogenesis pathways, this current knowledge can be hardly used to fully predict ribosome biogenesis in archaea (7,8). Moreover, a better understanding of ribosome biogenesis and function across the different domains of life is critical to unraveling the possible evolutionary trajectories and the molecular adaptations of these processes and might also provide relevant insights to, for example, engineer synthetic and minimal cellular systems (9–11). Accordingly, ribosome biogenesis in archaea and other nonmodel organisms remains to be fully elucidated (9–11).

Ribosome biogenesis is a multistep process characterized by the interplay of various molecular activities. Among these, the precursor ribosomal RNA (pre-rRNA) containing the mature rRNAs flanked by spacer regions needs to be processed by ribonuclease (RNase) activities, chemically modified, properly folded and assembled with ribosomal proteins (1–7).

**Figure 1.**
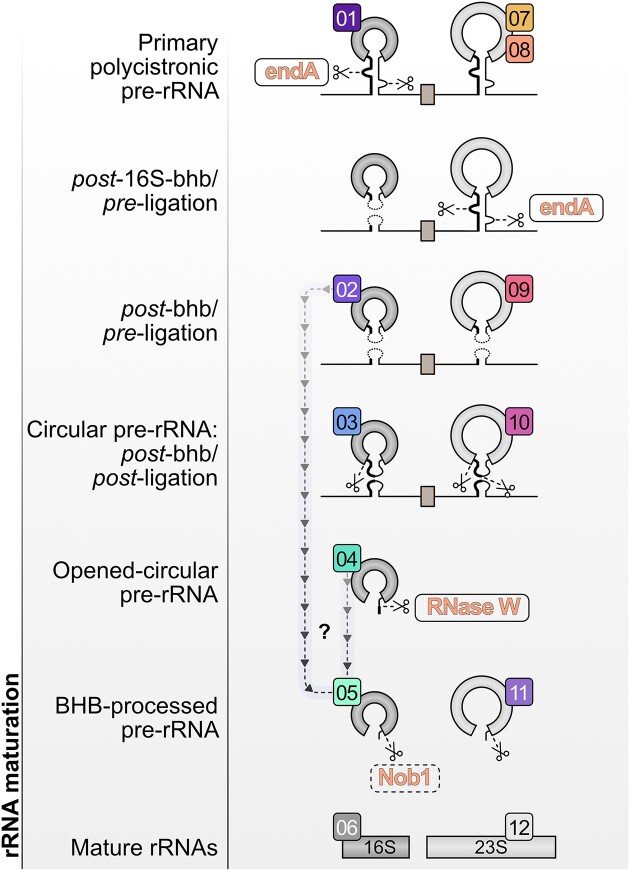
rRNA processing of an archetype archaeon, *Haloferax volcanii*. Updated rRNA processing pathway and known RNase activities (indicated by scissors) in *H. volcanii* are depicted. RNases for which *in vivo* and *in vitro* data are available are boxed, and RNases for which only *in vitro* data are available are indicated by a dashed box. Figure is modified from (14).

Recent studies have provided a comprehensive view of the archaeal rRNA maturation pathway and revealed common features shared among phylogenetically distant organisms as well as several specificities among the archaea studied (7,8,12–15) (Figure 1). Whereas most RNases involved in model bacterial and eukaryotic ribosome maturation pathways have been described, only a few RNase activities have been associated with the maturation of rRNAs in archaea thus far (16) (Figure 1). Among these RNases, the transfer RNA (tRNA) splicing endonuclease endA, in addition to its role in tRNA splicing, is involved in the maturation of the 16S and 23S rRNAs, whereby this RNase cleaves within the bulge–helix–bulge motif present in the flanking 16S and 23S rRNA processing stems (17). Following this, the resulting pre-rRNAs are covalently ligated at the resulting 5′ and 3′ ends akin to tRNA splicing (13,18,19). These maturation steps generate archaeal-specific pre-rRNA intermediates, the circular pre-16S (circ-pre-16S) and circ-pre-23S rRNAs, which are then further matured into linear rRNA intermediates (13,14,18,20) (Figure 1). In addition, the archaeal homolog of the endonuclease Nob1, an endonuclease involved in the maturation of the 3′ end extremity of the small ribosomal subunit in eukaryotes, has been shown, at least *in vitro*, to enable the maturation of the 16S rRNA 3′ end (13,21–23) (Figure 1). Finally, further *in vitro* analyses have demonstrated that the tRNase Z activity can also mature the 5′ end of the 5S rRNA, in addition to its role in tRNA maturation (24). However, based on the most recent description of the rRNA processing pathway in exemplary archaea, substantial discoveries remain to be accomplished to determine the full set of RNase activities involved in the archaeal rRNA maturation pathway *in vivo* (7,12,14) (Figure 1).

Several bacterial and eukaryotic RNase homologs have been identified by sequence homology in archaea, some of which are involved in rRNA processing in these domains of life (7,8,16). However, only a few have been characterized to some extent in archaea. Noteworthy and before the availability of several sequenced archaeal genomes, a study provided biochemical and serological evidence for an archaeal RNase G/E-like activity. This report provided immunogenicity and G/E-like catalytic activity of archaeal cellular extracts on a bacterial RNase G/E model substrate, the OmpA 5′UTR, thereby suggesting the presence of an RNase G/E-like activity in halophilic archaea (25). Interestingly, and in addition to its classical role in RNA degradation, the RNase G/E family members are also involved in the maturation of 16S rRNA in *Escherichia coli*, whereas this enzyme is absent in most Gram-positive bacteria, such as *Bacillus subtilis* (26–29). In archaea, a fusion protein containing sequences of RNase G/E-like homologs can be broadly detected (Supplementary Figure S1). However, these sequences lack several canonical features of this RNase family. For instance, the catalytic residues/domains involved in RNA maturation/degradation in bacteria are not well conserved (or absent) in the putative archaeal homologs (Supplementary Figure S1). Finally, in archaea, the G/E-like domain appears to be fused with another domain of unknown function (DUF402, previously annotated as a diphosphatase domain). However, the function of this protein domain remains elusive in archaea. Altogether the peculiar domain organization and sequence diversity observed for these archaeal G/E-like–DUF402 hybrid raised the question of whether this archaeal-specific protein domain organization is potentially active in RNA maturation/degradation and/or involved in 16S rRNA maturation, similar to its bacterial counterpart. Recently and in the course of performing this study, a *Pyrococcus furiosus* protein (named FAU-1, for *furiosus* AU-rich binding protein) that belongs to the archaeal G/E-like–DUF402 hybrid protein family has been shown to act as an endonuclease *in vitro* and its activity has been associated with 5S rRNA maturation (30,31). These findings potentially indicated that akin to the bacterial family, the archaeal G/E-like–DUF402 hybrid protein family might fulfil some function in rRNA processing. However, considering the absence of a genuine G/E-like catalytic center within this hybrid protein, the molecular basis for this RNase activity and its requirement for rRNA maturation in phylogenetically divergent archaea remained to be fully elucidated (30,31).

To shed additional light on the archaeal G/E-like–DUF402 hybrid protein family, we have performed a structure–function analysis. Our study provides structural and functional evidence that the archaeal DUF402 domain acts as an RNase, whereas the G/E-like domain is catalytically inactive, and may serve as a regulatory and substrate recognition subunit. We also provide *in vivo* evidence for its involvement in 16S rRNA maturation in *H. volcanii*. Based on our results, we provide an update of an archetype rRNA maturation pathway in archaea. Moreover, our study demonstrates that the archaeal G/E-like–DUF402 hybrid protein family defines a new group of RNases, which we termed the RNase W family. Our study further expands our knowledge of archaeal ribosome biogenesis and on functional diversification of RNase activity.

## Materials and methods

### Strains and plasmids

The open reading frames of RNase W (FAU-1) from *P. furiosus* (full-length protein, constructs and mutants; PF0022) and *S. acidocadarius* (Saci_1213) were synthesized commercially by GenScript Corp. (Piscatawy, NJ) and inserted in pET24(a+) (Novagen), pET24-*Pf*RNase W and pET24-*Sa*RNase W, respectively. The pET24-RNase W constructs contain an N-terminal 6xHis-tagged RNase W fusion protein. *Pf*RNase W constructs and mutants were designed: wild-type (WT) protein *Pf*RNase W (1–480); mutants in the central domain: RNase W-HH: H271A/H272A (1–480) and RNase W-RR: R10D/R12D (1–480); construct containing the C-terminal putative phosphatase domain: RNase W-Ct (325–480); and its mutants: RNase W-Ct-H326A and RNase W-Ct-D424K (325–480). Selected equivalent constructs of *Hv*RNase W-WT (HVO_0406) and mutants were cloned in *H. volcanii* expression vector pTA927 (32), generating pTA927-*Hv*RNase W, WT proteins (fused to an N-terminal or a C-terminal 3x Flag tag), and mutants R4A/R6D, H274D/H275D, H324A, D421R, C-terminal putative phosphatase domain only and N-terminal domain (lacking the putative phosphatase domain), all fused to a C-terminal 3x Flag tag.

### Recombinant protein expression and purification

The constructs were expressed in BL21DE3 strain from *E. coli* (Invitrogen) at 37°C in LB medium (Sigma–Aldrich) supplemented with kanamycin (100 μg ml^−1^) until OD_600nm_ between 0.6 and 0.8. Recombinant protein expression was induced by the addition of 0.5 mM isopropyl β-d-1-thiogalactopyranoside. Cell cultures were incubated for 4 h 30 min at 37°C and then harvested by centrifugation. Pellets were resuspended in buffer A (20 mM Tris–HCl, pH 7.5, 200 mM KCl) supplemented with protease inhibitors (Roche). Cells were lysed by sonication and the lysate was centrifuged for 20 min at 25 000 rpm (rotor JA-25.50, Beckman Coulter). For the crystallography experiment, an additional step was added consisting of incubating the cleared lysate with DNase I (Thermo Fisher Scientific) and RNase A (Thermo Fisher Scientific) at 37°C for 30 min. Then, the lysate was incubated at 65°C for 20 min and centrifuged for 20 min at 25 000 rpm (rotor JA-25.50, Beckman Coulter). These two last steps were skipped for the purified recombinant proteins used for the RNase activity assays. The cleared lysate was incubated for 1 h onto 5 ml of Ni^2+^-NTA resin (Thermo Fisher Scientific) at 4°C. Nonspecific proteins were removed by washing two times the resin with 10 ml of buffer B (20 mM Tris–HCl, pH 7.5, 200 mM KCl, 40 mM imidazole). The RNase W His-tagged protein was then eluted with 5 ml of buffer C (20 mM Tris–HCl, pH 7.5, 200 mM KCl, 400 mM imidazole), five times. The five eluted fractions were pooled, and the purified proteins were separated by gel filtration on a Superdex 75 16/60 (GE Healthcare) using buffer D [Tris–HCl, pH 7.5, 200 mM KCl, 1 mM dithiothreitol (DTT)].

### Crystallization, data collection and processing

The crystallization trials were performed using the hanging-drop vapor diffusion technique in 1 μl drops (with a 1:1 protein:precipitant ratio) equilibrated against 500 μl reservoir solution at 18°C. The *Pf*RNase W crystals from *P. furiosus* were obtained in 200 mM trisodium citrate, 25% (w/v) polyethylene glycol 3350 with the *Pf*RNase W protein at 8 mg ml^−1^ supplemented with 1 mM ADP (Adenosine DiPhosphate) and 5 mM MgCl_2_. The *Sa*RNase W crystal was obtained in 100 mM HEPES, pH 7.5, 20 mM MgCl_2_ and 22% polyacrylic acid (5100) sodium salt with the *Sa*RNase W protein at 5 mg ml^−1^ supplemented with 1 mM ADP and 5 mM MgCl_2_. Crystals were cryoprotected using three successive soaking steps with the reservoir solution containing 6.25%, 12.5% and 25% (w/v) polyethylene glycol 400. X-ray data were collected at the Soleil Synchrotron (Saint-Aubin, France) on Beamlines Proxima1 and Proxima2. For phasing, *P. furiosus* RNase W crystals were soaked in a solution composed of the reservoir solution supplemented with 10 mM potassium tetrachloroplatinate(II). Data were collected at the absorption threshold of platinum (1.0716 Å). Native and SAD datasets were indexed using the program XDS (33), and experimental phasing was carried out with the program Autosol (34) from PHENIX (35). Initial rebuilding was done with Resolve from the Autobuild module (36) from PHENIX and subsequent rebuilding and refinement with COOT (37) and the Refine module (37) from PHENIX. The dinucleotide, in the 5′-sensor domain, was built using structural homology from the RNase E-13-mer RNA complex structure from *E. coli* (PDB: 2C0B) (38). The dinucleotide in the phosphatase domain was built *de novo* with COOT (37). Molecular replacement, refinement, autobuilding and final refinement for the *Sulfolobus acidocaldarius* RNase W structure were carried out with Molrep (39), Buster (40), Arp/wArp (41) and PHENIX.REFINE (37), respectively.

### Purification and radiolabeling of bacterial RNA co-purified with *P. furiosus* RNase W

To purify the bacteria-associated RNA, we performed isolation of total RNA from *P. furiosus* RNase W sample purified as a recombinant protein in *E. coli* (100 μl coming from 1 l of culture). The sample was mixed with 200 μl of GTC lysis buffer [4 M guanidine thiocyanate, 2% sarkosyl, 50 mM Tris–HCl, pH 7.6, 10 mM ethylenediaminetetraacetic acid (EDTA) and 1% β-mercaptoethanol], 150 μl of sodium acetate buffer (10 mM Tris–HCl, pH 8, 1 mM EDTA, pH 8, 100 mM NaAc) and 450 μl of phenol:chloroform (1:1) and then incubated for 5 min at 65°C, followed by 5 min at 4°C and 3 min vortexing before centrifugation at 13 500 × *g* for 5 min at 4°C. The aqueous phase (250 μl) was transferred into a new sterile centrifuge tube and was then mixed with a triple volume of ice-cold 100% ethanol and the mixture was stored at −20°C for 2 h to precipitate RNA. The precipitate was collected by centrifugation at 14 500 × *g* for 30 min and washed in 70% ethanol. Then, it was again centrifuged at 14 500 × *g* for 15 min, ethanol was removed and the pellet was dried under vacuum before being resuspended in 70% formamide. The co-purified RNA was a mix of different sizes starting with a maximum of 100 nucleotides (nt). The co-purified RNAs were then separated on denaturing gel (8 M urea, 10% polyacrylamide) and only the largest fragment corresponding to the 100 nt identified by ultraviolet shadowing was excised and purified. Purified RNA was ^32^P labeled at the 5′ end and stored at −20°C until to be used.

### RNase *in vitro* activity assays

The 5 fmol of RNA was incubated with *Pf*RNase W-WT and mutant proteins (4 μmol) at 65°C in the presence or absence of Mg^2+^ (0.5 mM) in 20 μl of reaction buffer (20 mM HEPES, pH 8.0, 100 mM KCl). To stop the reaction, a mix of GTC/phenol/chloroform solution was immediately added to the reaction mixture, and the sample was transferred to ice. All RNAs from the samples were extracted by GTC/phenol/chloroform protocol as described for RNA purification (see above) and were separated on a 0.5× TBE (Tris Borate EDTA), 8 M Urea 10% polyacrylamide gel. For sample preparation, a gel loading mix (90% formamide, 0.5% EDTA, 0.1% xylene cyanol and 0.1% bromophenol blue) was added to the samples (50% final formamide concentration). Samples were heated for 2 min at 85°C before being loaded on the gel. After 1.5 h of migration (30 mA, 25 W), gels were carefully transferred into a dish with fixation solution (0.5× TBE, 10% acetic acid, 30% ethanol) for 5 min. The gels were vacuum-dried and exposed to a phosphoimager screen. The resulting autoradiography was scanned on a phosphoimager (BAS5000, GE Healthcare).

### Growth and genetic manipulation of *H. volcanii*

*Haloferax volcanii* strains were grown aerobically in YPC (Yeast extract with Permeate and Citrate) medium or HvCa+ (Enhanced casamino acids medium for *H. volcanii*) selective medium at 42°C under vigorous agitation as described previously (19,42–44). H26 and its derivative H119 were used as WT strains (44). Markerless deletion strain ΔHVO_0406 was generated as described (45). In short, the open reading frame for *Hv*RNase W was deleted except for the first four nucleotides, that overlap with the upstream gene HVO_0407. H119 was transformed with the deletion plasmid pTA131-UPDO(*rnGE*) using the PEG600 protocol (46). Deletion strains were obtained using the pop-in/pop-out method (47). Knockout candidates were identified by polymerase chain reaction (PCR) and homozygous deletion strains were subsequently confirmed via Southern blot analysis.

### rRNA maturation analysis in *H. volcanii*

Total RNAs were extracted using RNA extraction kits from Macherey-Nagel (NucleoSpin RNA) or Analytik Jena (innuPREP Micro RNA Kit). Two to three micrograms of total RNA were separated by denaturing agarose gel electrophoresis followed by northern blotting analysis as described previously (14). Pairwise hybridization (DY682/DY782 label combination) with the following fluorescently labeled probes was carried out overnight at 30°C after at least 3 h of prehybridization of membranes: Probe 1, targeting the mature 16S rRNA (oHv707: DY682-GAGCTGGTGAGATGTCCGGC); Probe 2, targeting the 16S ligation site (oHv762: DY782-CCGTTCGGATaaggtgtccg); Probe 3, targeting the 16S 3′ trailer (oHv325: DY782-CGTGTGAGCCACCCCGTCGG); and Probe 4, targeting mature 23S rRNA (oHv544: DY682-TGTGCTCTTGCCGGTGACGA). For the detection of mature rRNAs (DY682 label) or pre-rRNAs (DY782 label), 1–2 or 25–100 pmol per hybridization reaction were used, respectively. After hybridization membranes were washed once with 2× SSC and once with 1× SSC (Saline Sodium Citrate) buffer for 15 min at 30°C. Detection of fluorescent signals was carried out on a Li-Cor Odyssey imaging system at 700 nm (DY682 label) and 800 nm (DY782 label).

RNase H-dependent cleavage assays were performed according to Grünberger *et al.* (14). In short, two to three micrograms of total RNAs were incubated with primers A (oHv203: AGGAGGTGATCCAGCCGC) and B (oHv254: GTACTTCCCAGGCGGCTCG) (5 pmol each) and 1.5 μl 10× RNase H buffer (1× RNase H buffer: 50 mM Tris–HCl, pH 8.3, 75 mM KCl, 3 mM MgCl_2_,10 mM DTT) for 5 min at 65°C followed by oligo annealing for 10 min at 37°C in a total volume of 14 μl in a PCR cup. Five units of RNase H (NEB) were added, followed by an incubation of 20 min at 37°C. Fifteen microliters of 2× formamide buffer [0.05% (w/v) bromophenol blue in 100% formamide] was added and the reaction stopped at 65°C for 15 min. The RNase H cleaved total RNAs were loaded on a denaturing agarose gel and further analyzed by northern blotting as indicated earlier and in (14).

## Results

### Overall structure of the RNase W protein family

To obtain additional insights into the putative function and domain architecture of the archaeal G/E-like–DUF402 hybrid protein family, the full-length proteins from the archaea *P. furiosus* (hereafter, *Pf*RNase W) and *S. acidocaldarius* (hereafter, *Sa*RNase W) were expressed in a bacterial system. A six-histidine tag was fused to the N-terminus of the proteins for a first purification step by nickel affinity, followed by size exclusion chromatography. Since both *Pf*RNase W and *Sa*RNase W proteins were co-purified with contaminant bacterial RNA, an RNase treatment was performed on the cell lysis supernatant before the two-step purification procedure. The structure of the *Pf*RNase W protein was solved by single isomorphous replacement with anomalous scattering using platinum-derived and native crystal diffracting to 2.6 and 2.1 Å, respectively. The structure of the *Sa*RNase W protein was solved to 2.0 Å by molecular replacement using the *Pf*RNase W protein as a model. One copy of RNase W protein is present in the asymmetric unit in both space groups. The final models for both RNase W proteins include all the amino acids except for the four N-terminal residues and the histidine tag. Two magnesium ions, two phosphate ions and two bound dinucleotides from the co-purified RNA are visible in the *Pf*RNase W structure and only two magnesium ions in the *Sa*RNase W structure. Data collection and refinement statistics are reported in Table 1.

**Table 1.** X-ray diffraction and refinement statistics

Data collection			
	*P. furiosus*		*S. acidocaldarius*
	Native	PtCl_4_	Native
Wavelength (Å)	0.97	1.07	0.97
Resolution	32.6–2.1	42.8–2.6	28.3–2.0
Space group	*P*321	*P*321	*P*41
Unit cell (Å)	120.6, 120.6, 93.8	120.7, 120.7, 94.0	76.2, 76.2, 84.6
Total reflections	937 845 (94 497)	975 011 (91 587)	138 075 (13 244)
Unique reflections	46 329 (4588)	24 008 (2280)	30 604 (3052)
Multiplicity	20.2 (20.6)	40.6 (40.2)	4.5 (4.3)
Completeness (%)	99.9 (100.0)	99.6 (97.1)	99.6 (99.4)
Mean *I*/*σ*(*I*)	17.8 (1.5)	34.9 (4.7)	8.0 (1.1)
*R*-merge (%)	11.2 (193.3)	10.0 (87.9)	13.9 (117.7)
CC 1/2	0.9 (0.6)	1 (0.9)	0.9 (0.4)
CC*	1 (0.8)	1 (0.9)	0.99 (0.8)
**Model refinement**			
	***Pf*RNase W**	***Sa*RNase W**	
PDB ID			
*R*-work	0.21 (0.29)	0.18 (0.26)	
*R*-free	0.24 (0.34)	0.21 (0.29)	
Nonhydrogen atoms	4085	3643	
Macromolecules	30 857	3388	
Ligands	35	2	
Protein residues	465	415	
RNA bases	4		
RMS deviations			
Bond lengths (Å)	0.007	0.013	
Bond angles (°)	1.15	1.45	
Ramachandran plot			
Favored (%)	97.4	97.6	
Allowed (%)	2.4	2.4	
Outliers (%)	0.2	0	
Rotamers outliers (%)	0	2.65	
Clashscore (all atoms)	5.25	6.16	
Average *B*-factor	55.41		
Protein	55.11	35.29	
Ligands	67.96	30.00	

**Figure 2.**
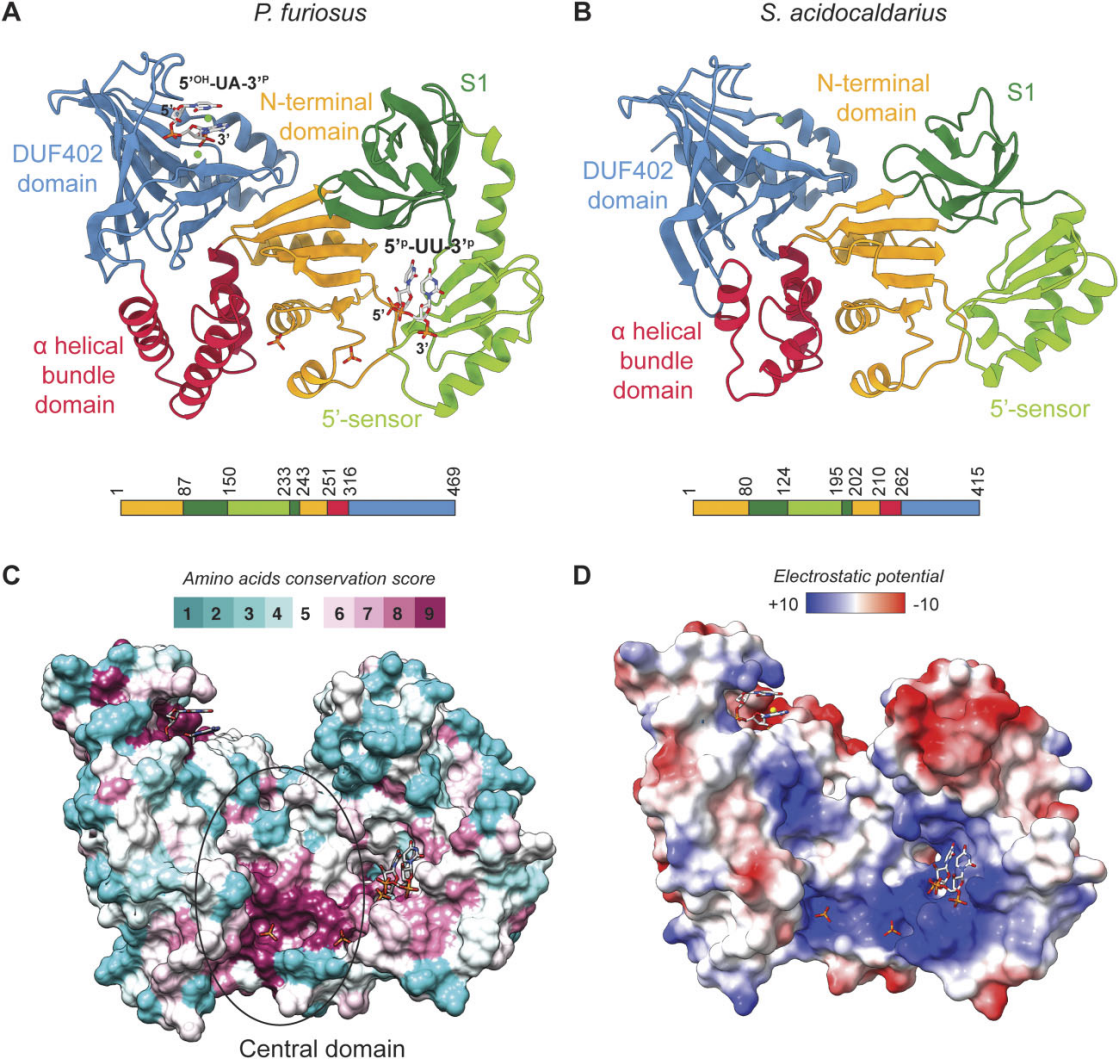
Structures of RNase W from *P. furiosus* and *S. acidocaldarius*. (**A** and **B**) Structures of RNase W proteins colored by domains: N-terminal domain in orange, S1 domain in dark green, 5′-sensor domain in light green, α helical bundle domain in red and DUF402 domain in blue. (**A**) Structure of RNase W from *P. furiosus* (*Pf*RNase W). A dinucleotide UU with 5′ and 3′-phosphate extremities is bound to the 5′-sensor domain. In addition, a dinucleotide UA with 5′-hydroxyl and 3′-phosphate extremities is bound to the DUF402 domain. (**B**) Structure of RNase W from *S. acidocaldarius* (*Sa*RNase W). (**C**) *Pf*RNase W surface representation with residues conservation score in archaeal RNase W proteins mapped on the surface. The encircled central domain includes the N-terminal domain and one helix of the α helical bundle domain annotated in panel A. (**D**) Electrostatic surface potential mapped on the surface of *Pf*RNase W protein.

The proteins adopt a butterfly-shaped structure and can be decomposed in five domains: an N-terminal domain at the center (orange in Figure 2A and B), S1 and 5′-sensor domains inserted in a loop between two β strands (dark and light green, respectively) and at the C-terminus an α helical bundle (red) followed by a putative phosphatase domain (DUF402) (blue). Although the overall structures of both RNase W proteins are similar with 27.5% sequence identity, they can be superimposed with a 5.6 Å root mean square deviation (rmsd) over all 400 C-alpha atom pairs (Figure 2A and B). The domain orientations only vary slightly between both proteins; each domain can be superposed individually with an ∼1 Å rmsd on equivalent residues (across 58 atom pairs for N-terminal domain, across 6 atom pairs for S1 domain, across 53 atom pairs for 5′-sensor domain, across 33 atom pairs for α helical bundle domain and across 110 atom pairs for DUF402 domain). Due to this similarity, in the rest of the manuscript, unless stated otherwise, the *Pf*RNase W structure will be used for the structural analysis.

The sequence conservation score of amino acids for homologous archaeal RNase W proteins mapped on the surface of the protein (48) highlights two very conserved regions: one in the large central domain between the N-terminal and 5′-sensor domains and one in the putative phosphatase domain (Figure 2C). This highlights two important sites in the protein that also coincide with the observed RNA fragments bound in the *Pf*RNase W structure (see further). The surface of the protein is composed of a large positive region, including the 5′-sensor domain, the central domain and the putative phosphatase domain. These positive regions could bind RNA as suggested with the RNA fragments and phosphates bound in the *Pf*RNase W structure (Figure 2D). Multiple sequence alignments on these conserved regions are shown in Supplementary Figure S2.

### RNase W contains an RNase E-like S1 and 5′-sensor domains

**Figure 3.**
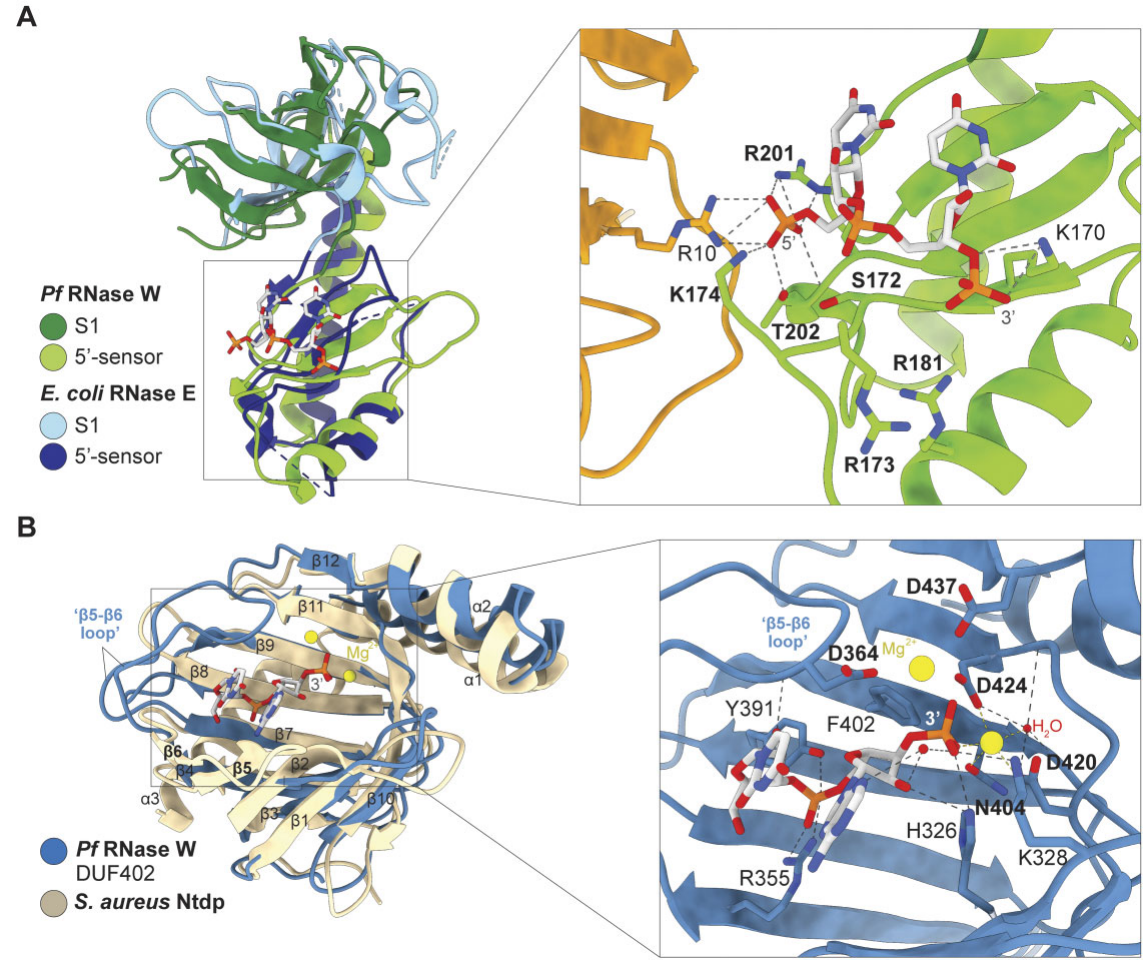
Structural homologies and analysis of ligand interactions with *Pf*RNase W. (**A**) Superposition of RNase E domains (in light and dark blue, PDB ID 2C0B) on *Pf*RNase W (in light and dark green) in the left panel. The dinucleotide UU is shown in light gray. Identical or equivalent residues with the 5′-sensor domain of RNase E from *E. coli* are indicated in bold. (**B**) Superposition of structural homolog Ntdp nucleoside tri- and diphosphatase from *Staphylococcus aureus* (in beige, PDB ID 7D8L) to *Pf*RNase W DUF402 domain (in blue) in the left panel. A dinucleotide UA with a 3′-phosphate end (in light gray) is bound in the central cavity of the *Pf*RNase W domain as well as two magnesium ions. All shown residues in the right panel are conserved among Archaea. In bold, the equivalent residues present in all the structural homologs of the RNase W putative phosphatase domain are indicated.

The G/E-like–DUF402 hybrid protein family has been classified as putative RNase G/E homolog due to the presence of a domain with sequence similarity with the S1 and 5′-sensor domains. The S1 and 5′-sensor domains of *Pf*RNase W and *Sa*RNase W present 20.3% and 10.9% sequence identity with the corresponding domains of *E. coli* RNase E (RNase E) and can be superimposed with 2.5 and 3.7 Å rmsd, respectively, with the *E. coli* RNase E structure (Figure 3) (38). In RNase E, these two domains interact with RNA and participate in RNA recognition. The RNase E 5′-sensor domain specifically binds the 5′-phosphate of the substrate RNA, and the S1 domain extends the binding interface, which provides a total binding interface for ∼10-mer single-stranded RNA. This is used by RNase E to specifically recognize short single-stranded RNA with a 5′-phosphate and position the RNA in the enzyme’s active site through a conformational reorganization (29,38).

In the *Pf*RNase W structure, a dinucleotide is bound in the 5′-sensor domain (Figures 2A and 3, and Supplementary Figure S3A). The type of nucleic acid bases in the dinucleotide is not clearly identifiable from the electron density and possibly corresponds to a mixture of different nucleotides bound to the proteins in the crystal, but since pyrimidine bases gave a better fit to the observed densities, the dinucleotide was modeled with uracil bases.

Remarkably, the bound dinucleotide has both 3′- and 5′-phosphate extremities visible in the electron density. The presence of a dinucleotide is thought to arise from the generation of a stably bound fragment from bacteria-associated RNA co-purified with the protein and digested by the RNase treatment during purification.

*Pf*RNase W interacts mainly with the dinucleotide phosphate moieties with basic amino acids present in the 5′-sensor domain that are conserved in archaea (Figures 2C and D, and 3A and B). K170, R173 and R181 form a strong basic patch interacting with the 3′-phosphate of the second nucleotide. R201, T202 and K174 interact with the 5′-phosphate extremity of the first nucleotide through salt bridges and hydrogen bonding network involving all three phosphate oxygens. S172 also participates in this network as well as R10 from the central N-terminal domain.

RNase E has been crystallized with di- or trinucleotides, similarly bound from the exogeneous RNA during expression and purification, and with a 13-mer RNA (PDB ID 2C0B, 2VRT, 5F6C) (38,49,50). Comparison of the *Pf*RNase W structure with the RNase E from *E. coli* structures bound to RNA reveals that the binding mode of the 5′-phosphate is conserved with identical or equivalent residues at positions R201 (R169), T202 (T170), S172 (S140) and K174 (R142) (*E. coli* numbering in parenthesis), and with identical residues for 3′-phosphate binding at R173 (R141) and R181 (R148) (Figure 3B) (38). This clearly indicates that similarly to RNase E, the 5′-sensor domain of *Pf*RNase W has all the required molecular and structural properties to function in specific recognition of small single-stranded RNAs containing a 5′-phosphate. In RNase E, the positioning of the S1 domain provides an extended interaction surface that can accommodate a 13-mer single-stranded RNA (38). Modeling of the RNase E bound 13-mer RNA in the *Pf*RNase W structure by structural superposition seems to indicate that the *Pf*RNase W S1 domain together with the 5′-sensor domain also has the capability to bind to single-stranded RNA of ∼13 nt (Supplementary Figure S4A).

The structural homology and the presence of bound RNA interacting with conserved residues confirms the presence of a functional RNAse G/E-like domain in RNase W protein. This homology with RNase E is, however, restricted to the 5′-sensor and S1 domains that function in the specific binding of short 5′-phosphate containing single-stranded RNAs. The other domains of *Pf*RNase W do not share any homology with the RNase E domains, notably the absence of an RNase E-like catalytic domain.

### RNase W contains a divergent DUF402 domain

The C-terminal domain of *Pf*RNase W (*Pf*RNase W Ct) is annotated as belonging to the DUF402 domain phosphatase family. Our structure reveals that it is structurally homologous to proteins from this family with an rmsd of 2.4 Å and 16% sequence identity on average (51). Proteins from this family have been shown to have a nucleoside diphosphatase activity (51). The best characterized homologous structures are the Ntdp (Nucleoside tri- and diphosphatase) from *S. aureus* involved in bacterial virulence (51), the FomD cytidylyl (*S*)-2-hydroxypropylphosphonate hydrolase involved in fosfomycin biosynthesis (52) and the SC4828 phosphatase from *Streptomyces coelicolor* (PDB 3EXM) (Supplementary Figure S5). All these enzymes, collectively referred to as the DUF402 domain below, have a common enzymatic mechanism consisting of the hydrolysis of nucleotide di- or triphosphates to nucleotide monophosphates. It is not yet clearly defined whether the enzyme sequentially cleaves one phosphate at a time or can directly cleave between the α and β phosphates of nucleotide triphosphates.

The putative phosphatase domain of *Pf*RNase W shares the common fold with its structural homolog, consisting of a 12 β-strand N-terminal β-barrel domain and three C-terminal helices (Figure 3A, and Supplementary Figure S4B). The β barrel is composed of two antiparallel β sheets, containing the β10-1-2 strands and the β3-4-7-8-9-11-12 strands, respectively. Contrarily to the DUF402 family, where β5 and β6 extend the first β sheet after β2, the β5 and β6 strands in *Pf*RNase W form a β loop that packs against the second β sheet to form a larger groove in the central cleft (Figure 3A, and Supplementary Figure S4B).

Ntdp and SC4828 proteins have been crystallized with nonhydrolyzable nucleotide di- or triphosphates, and FomD with nucleotide diphosphate, shedding light on substrate binding mode and catalytic mechanism (51,52). The central cleft between the β sheets comprises the ligand binding site. Two metal ions define the enzymes active site. A network of acidic residues from β10–β11 loop, the β11 strand and the α1 helix coordinate the two metal ions and the terminal phosphate, positioning the nucleotide’s terminal 5′-phosphate for inline attack. Most of these residues are conserved in the RNase W putative phosphatase domain (see further). The suggested catalytic mechanism involves the activation of a water molecule by an aspartate to promote a nucleophilic attack on the terminal phosphonate group (Supplementary Figure S4B and C) (51). The metal ion then activates the phosphonate as a Lewis acid, the substrate phosphate group is cleaved and an arginine acts as a proton source.

Surprisingly, a dinucleotide is present in the center of the putative phosphatase domain of *Pf*RNase W (Figure 2A). The electron density of the dinucleotide bases clearly showed a purine base followed by a pyrimidine base, which we have modeled as 5′-UA-3′ with the 3′-phosphates clearly visible in the density (Supplementary Figure S3B). Similarly to the dinucleotide in the 5′-sensor domain, this dinucleotide was bound from the bacteria-associated RNA digested by the RNase treatment during purification.

In spite of strong structural homologies to the DUF402 domain phosphatase family, the presence of the fortunately bound ligand in the structure sets the RNase W putative phosphatase domain apart from its bacterial homologs. First, the bound dinucleotide indicates that it has a different substrate specificity. Moreover, the dinucleotide presents its 3′-phosphate in the enzyme’s active site, in the same position where the 5′-phosphate of the nucleotides bound in DUF402 phosphatases are found (Figure 3B, and Supplementary Figures S4B and C and S5) (51,52). The RNase W substrate is therefore bound in a reverse polarity compared with the structurally homologous DUF402 dinucleotide phosphatases (Supplementary Figure S5).

Despite the difference in bound substrate, the active site of the enzyme is remarkably similar. Three residues, D420, D424 and N404 in *Pf*RNase W, are universally conserved in all the structural homologs and coordinate a magnesium ion involved in binding the substrate terminal phosphate (Figure 3B). RNase W, however, lacks a second magnesium present in all DUF402 phosphatases and coordinated by residues in equivalent positions to D424 and D437. In *Pf*RNase W, the third aspartate coordinating this magnesium is replaced by a lysine, which could explain the absence of a magnesium binding site. Instead, a second magnesium is found in proximity, coordinated by D424, D437 and D364 from the β5–β6 loop. The interaction with D364 is only possible due to the different conformation of the *Pf*RNase W β5–β6 loop compared with DUF402 homologs, which is required to place D364 in position to coordinate the magnesium (Figure 3B).

In addition to the magnesium atoms and the coordinating aspartates, the terminal 3′-phosphate is bound by K328 and H326, which are potentially important residues for the domain’s catalytic activity (Figure 3B). The 5′-phosphate of the dinucleotide is bound by R355, Y391 and T378. The structure of the *Pf*RNase W putative phosphatase domain with the unique conformation of the β5–β6 loop creates a narrow groove that can accommodate several nucleotides at the 5′ and 3′ ends. It is therefore probable that the natural substrate of the *Pf*RNase W putative phosphatase domain is a single-stranded RNA (Supplementary Figure S4D).

All residues previously described are conserved among Archaea (Figure 2C), suggesting their importance for the ligand interaction and probably for the function of the RNase W putative phosphatase domain. We hypothesize that the active site for the catalytic activity is centered on the 3′-phosphate of the dinucleotide. Although the substrate is different from the DUF402 domains, the catalytic site conserves the two magnesium ions that could be involved in the activation of a water molecule and the nucleophilic attack on the 3′-phosphate. K328 or H326 could then act as proton sources for completion of the reaction (Supplementary Figure S6). The nature of the catalysis performed cannot, however, be determined from the structure. A plausible hypothesis includes the endo- or exonucleolytic cleavage of RNA, cleavage of terminal 3′-phosphates or the resolution of cyclic 3′-phosphate. We have tested for phosphatase activity using a variety of substrates (RNA, di- or triphosphate nucleotides, etc.) and buffers but could not detect any activity (data not shown).

### The central domain does not harbor RNase activity

G/E-like–DUF402 hybrid proteins are annotated in databases as probable RNases but no sequence or structural homology with an RNase catalytic domain was found in our RNase W structures. The annotation comes from the presence of a 5′-sensor/S1 domain similar to the one found in the RNase G/E family despite the absence of a G/E-like catalytic domain. Besides the 5′-sensor and the putative phosphatase domains, RNase W contains a central domain that has no homology with known structures from the Protein Data Bank. Moreover, the central domain has residues highly conserved in Archaea, indicating an important domain for protein function (Figure 2C).

Therefore, we first hypothesized that this central domain could carry the potential RNase activity of RNase W protein. In the *Pf*RNase W structure, two phosphates from bacterial bound-RNA are present in the central domain (Figure 2, and Supplementary Figures S3C and D). Each phosphate binds basic residues: phosphate annotated P1 interacts with R40, R261, K275, T17 and P2 interacts with R12 and K45, both through hydrogen bonds. H271 binds P1 through the interaction with a molecule of water. These observations suggest arginine and lysine residues stabilize RNA so that the H271 triggers the cleavage reaction. In addition to previously cited residues, H272 is also a conserved amino acid among Archaea, suggesting their importance for RNA interaction and/or RNase activity.

**Figure 4.**
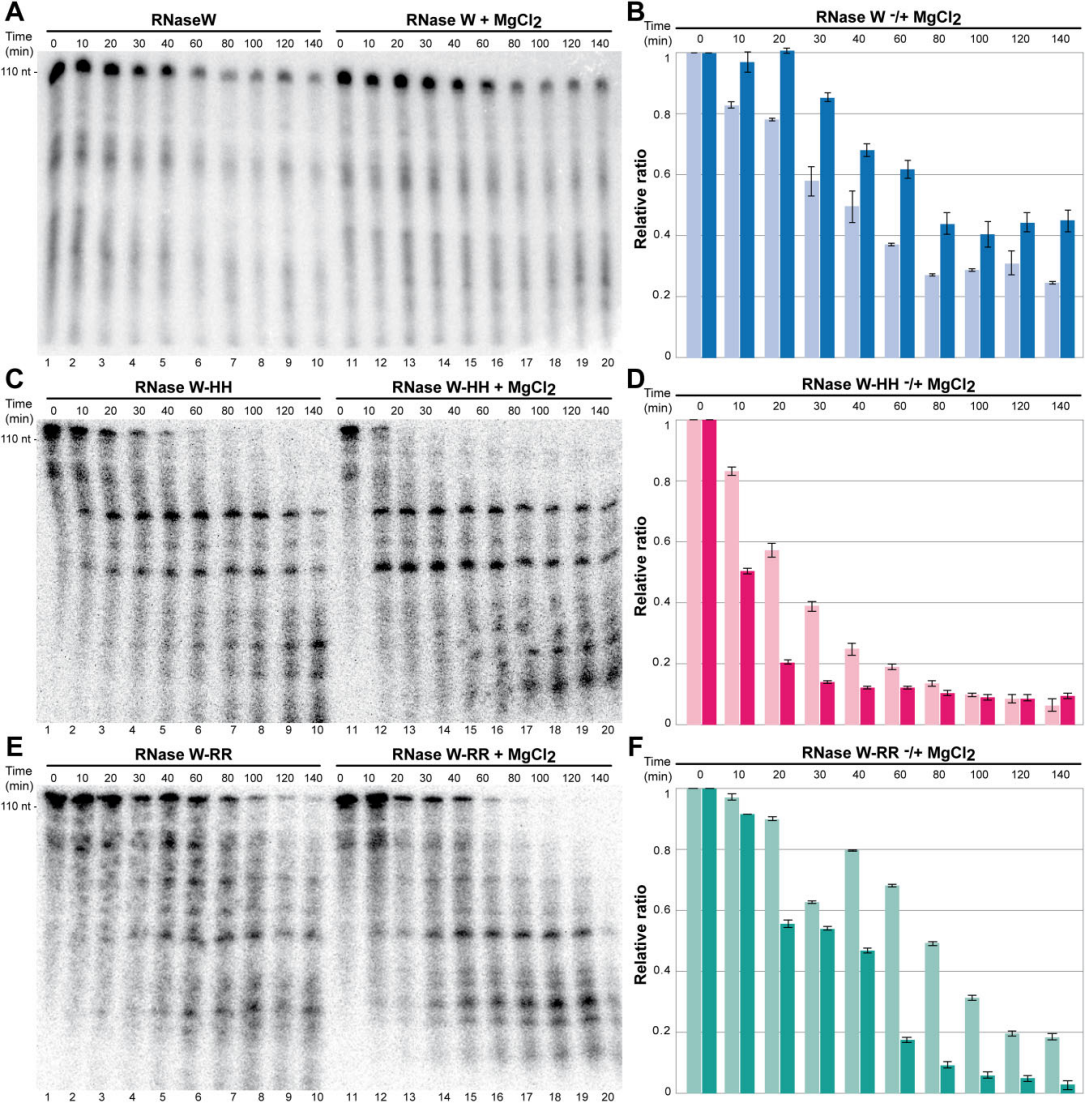
RNase activity assay of *Pf*RNase W constructs *in vitro*. *Pf*RNase W protein (4 μM) was incubated with 5 fmol of RNA (co-purified and radiolabeled) at 65°C over a time course (140 min) and then analyzed by 8 M urea 10% polyacrylamide gel. Comparison of RNase activity in the absence (wells 1–10) and in the presence (wells 10–20) of magnesium (MgCl_2_ at 0.5 mM) for (**A**) *Pf*RNase W-WT, (**C**) *Pf*RNase W-HH and (**E**) *Pf*RNase W-RR. Relative ratio calculated from the intensity of the top RNA band quantified four times and normalized to the time 0 intensity. RNAs were incubated with (**B**) *Pf*RNase W-WT (blue), (**D**) *Pf*RNase W-HH (pink) and (**F**) *Pf*RNase W-RR (green) constructs without (light colors) and with MgCl_2_ (dark colors).

**Figure 5.**
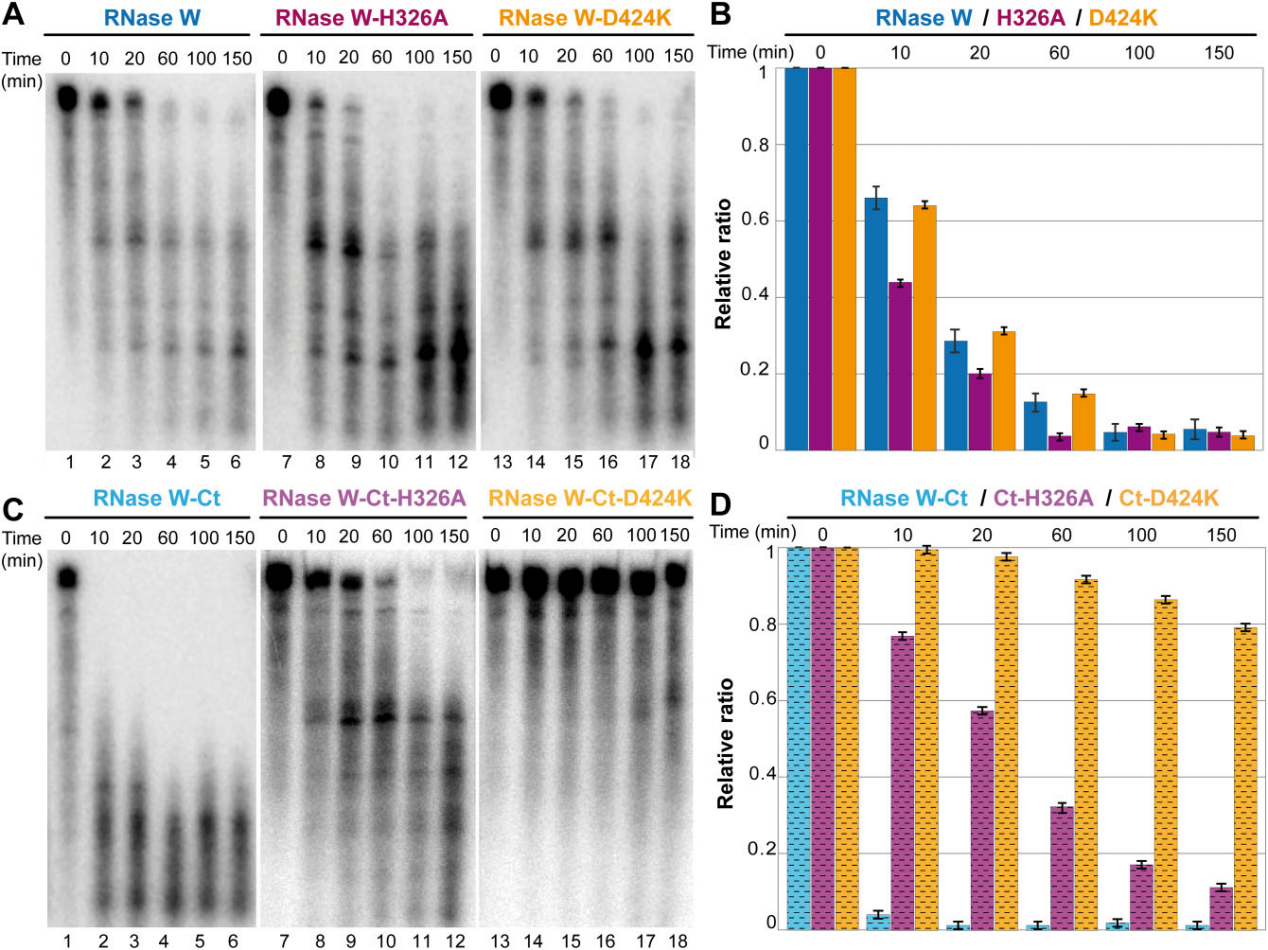
RNase activity of C-terminal domain of *Pf*RNase W and mutant proteins. RNase activity assay: mutant *Pf*RNase W proteins (4 μM) were incubated with 5 fmol of RNA (co-purified and radiolabeled) at 65°C in the presence of magnesium (MgCl_2_ at 0.5 mM) over a time course (120 min) and then analyzed by 8 M urea 10% polyacrylamide gel. (**A**) Gel analysis of RNase activity of full-length *Pf*RNase W proteins: *Pf*RNase W (lanes 1–6), *Pf*RNase W-H326A (wells 7–12) and *Pf*RNase W-D424K (lanes 13–18). (**B**) Relative ratio of the RNA incubated with WT *Pf*RNase W (dark blue) compared with H326A FL mutant (purple) and D424K FL mutant (orange) (quantified four times). (**C**) Gel analysis of RNase activity assays using C-terminal domain of *Pf*RNase W (lanes 1–6) compared with mutants in the potential catalytic site: H326A (lanes 7–12) and D424K (lanes 13–18). (**D**) Relative ratio of *Pf*RNase W-Ct (striped light blue) compared with *Pf*RNase W Ct-H326A (striped light purple) and *Pf*RNase W Ct-D424K (striped light orange) (quantified four times).

In order to test the nuclease activity and identify the catalytic site, we have performed *in vitro* nuclease assays with various *Pf*RNase W constructs and mutants (Figures 4 and 5). Sodium dodecyl sulfate–polyacrylamide gel electrophoresis analysis of the WT and mutant recombinant proteins showing their integrity is provided in Supplementary Figure S7. Because the native substrate of *Pf*RNase W is unknown, we took advantage of the fact that the protein heterologously expressed in *E. coli* co-purifies with bacterial RNA. We reasoned that this RNA could be a good substrate for *Pf*RNase W activity. We purified this RNA from bound *Pf*RNase W by phenol extraction and ethanol precipitation and radioactively labeled the 5′-phosphate. We then re-incubated this RNA at 65°C with purified *Pf*RNase W protein constructs or mutants and visualized the integrity of the RNA on denaturing polyacrylamide–urea gel at different time points.

The co-purified RNA exhibits distinctive band sizes, indicating that *Pf*RNase W preferentially binds an RNA with a size of ∼110 nt. Smaller fragments of 60 and 20 nt can be observed. During a 140-min time course, a progressive disappearance of the larger RNA band can be observed. In contrast, if magnesium is added to the reaction mixture, the RNA is stabilized, and less degradation is observed. WT *Pf*RNase W therefore shows RNase activity that is modulated by magnesium (Figure 4A).

To test the involvement of the central domain in nuclease activity, two constructs with the conserved histidine residues H271 and H272 mutated to alanine (*Pf*RNase W-HH) and the arginines R10 and R12 mutated to aspartate (*Pf*RNase W-RR) were tested in the RNA degradation assay. The addition of magnesium enhanced the RNase activity of these mutants (Figure 4B and C). Therefore, mutation of conserved residues in the central domain of *Pf*RNase W activates the RNase activity, which demonstrates that the central domain does not harbor the RNase catalytic site.

### The *Pf*RNase W DUF402 has RNase activity in RNAse W

Since the central domain did not harbor the RNase activity, we reasoned that the *Pf*RNase W Ct domain, which did not show phosphatase activity in our assays (data not shown), could be the RNase catalytic domain. We therefore generated two *Pf*RNase W constructs, *Pf*RNase W-D424K and *Pf*RNase W-H326A, where conserved residues in the proposed catalytic site were mutated (Figure 5A and B). Both these mutants retained an RNase activity comparable to the WT protein (Figure 5A and B).

The same experiments were performed with the *Pf*RNase W-Ct construct retaining only the DUF402 domain. Surprisingly the *Pf*RNase W-Ct domain degrades the RNA totally before the first 10 min time point (Figure 5C andD). This result indicates that contrarily to the other members of the DUF402 family, the RNase W DUF402 domain does not have nucleotide phosphatase activity but harbors potent RNase activity. In the context of the *Pf*RNase W-Ct construct, the catalytic site’s mutant H326A shows a severely reduced activity while the activity of the D424K mutant was completely abolished (Figure 5C and D), confirming the involvement of these residues in RNase activity. This is in contrast to the results of these mutants in the full-length protein construct, which retained an activity close to the WT protein indicating that the activity seen in the WT protein is probably regulated by the presence of the N-terminal domain (Figure 5A and B). We observed similar results in the absence of magnesium (Supplementary Figure S8).

In summary, the putative phosphatase domain harbors a potent RNase activity, only when it is isolated from the rest of the *Pf*RNase W domains. This seems to indicate that there exists an internal regulation mechanism that inhibits RNase activity in the context of the full-length protein. Finding an RNase activity in a domain usually associated with nucleotide diphosphatase activity is surprising, but the nature of the catalytic mechanism is similar and, in both cases, involves the cleavage of a phosphate diester bond (51). We have moreover confirmed that the catalytic residues important for phosphatase activity in the DUF402 family are essential for RNase activity in RNase W protein.

### A molecular switch for 5′-phosphate recognition

In RNase E, the 5′-sensor and S1 domains, respectively, function in the recognition of a 5′-phosphate on an AU-rich single-stranded RNA, allowing RNase E to select RNA substrates that are unstructured at their 5′ end, excluding the degradation of functional structured RNAs (29,38,50). The tetrameric arrangement of RNase E enables the 5′-sensor domain to position the RNA in line for cleavage by the catalytic site of an RNase catalytic domain of a second subunit (29,49).

However, unlike RNase E, *Pf*RNase W does not contain this type of RNase domain and is purified as a monomer (SEC and SAXS, data not shown), which indicates that the putative RNase activity is performed by a totally different mechanism. We have identified the C-terminal domain as the RNase domain and showed that the RNase activity is partially inhibited in the context of the full-length protein.

**Figure 6.**
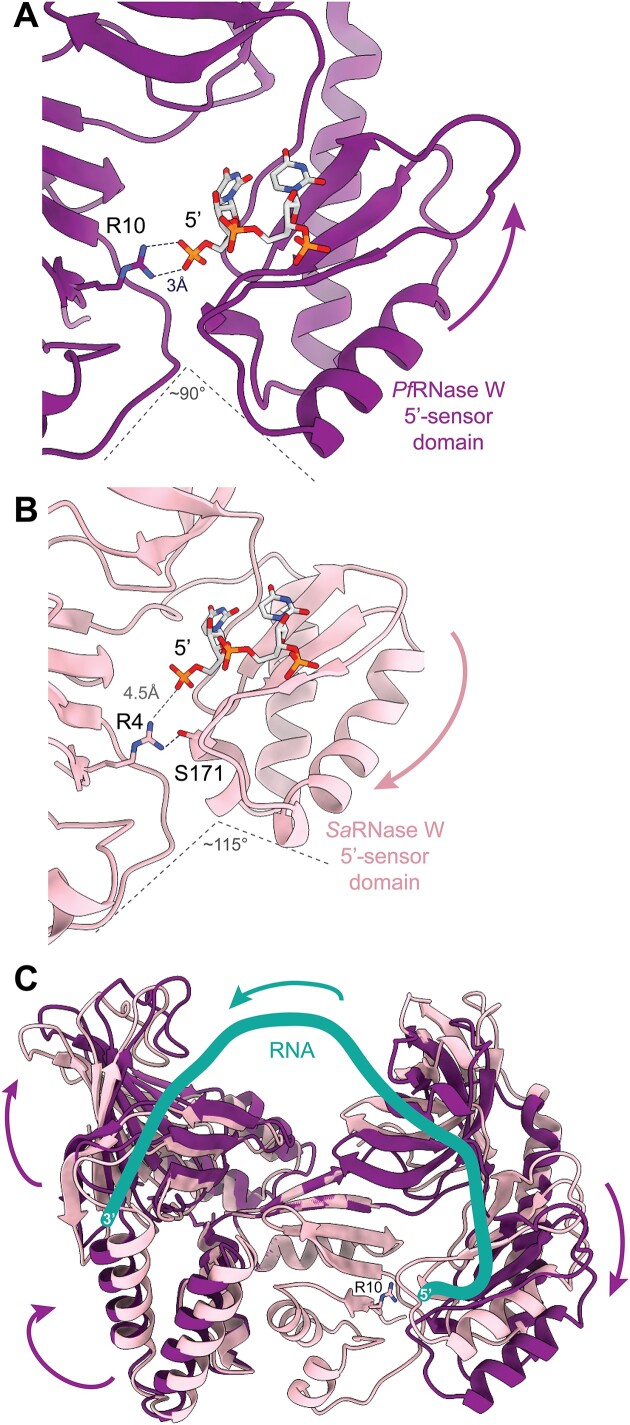
Conformational changes upon RNA binding. (A and B) Structure of RNase W protein focused on the 5′-sensor domain showing a molecular switch for 5′-phosphate recognition. (A, B and C) *Sa*RNase W and *Pf*RNase W proteins are superimposed on the central domain to highlight the movement of the other domains. (**A**) *Pf*RNase W protein (purple) with dinucleotide 5′-phosphate (in gray) bound in the 5′-sensor domain. R10 forms a double H bond (in blue dashed line) with the 5′-phosphate. Upon 5′-phosphate recognition, the 5′-sensor domain would move downward. Thus, the R10 (R4 in *Sa*RNase W) would lock the domain in a closed conformation. (**B**) *Sa*RNase W protein (light pink) with modeled dinucleotide 5′-phosphate (in gray) from the *Pf*RNase W structure, bound in the 5′-sensor domain. R4 forms an H bond (in blue dashed line) with S172 but the basic residue is ∼4.5 Å (in gray dashed line) away from the 5′-phosphate. R4 does not interact with 5′-phosphate. The arrow indicates the movement of the 5′-sensor domain during 5′-phosphate recognition. (**C**) Molecular representation of *Pf*RNase W structure in two states: apo (light pink) and RNA binding (pink). The apo state was modeled according to the structural conformation of *Sa*RNase W domains. The proposed pathway of RNA in the protein is drawn in green. The arrows indicate the movement of the different domains upon RNA binding. A movie showing the conformational changes upon RNA binding is available as Supplementary Video S1.

Comparison of the structures of the *P. furiosus* and *S. acidocaldarius* RNase W proteins (this work) shows that although the domain structures are similar, the overall relative conformation of the domains differs in the two proteins. Figure 6 shows the two proteins with the superposition on the central domain. This representation shows a large rotation of the 5′-sensor and S1 domains, and a smaller movement of the N-terminal domain (Figure 6C).

Although differences in domain orientation can arise from crystallization artefacts, we investigated whether this difference could be due to the binding of RNA fragments in *Pf*RNase W. Indeed, rotation of the 5′-sensor and S1 domains leads to different contacts with the central domain (Figure 6A and B). In the *Pf*RNase W structure with a 5′-phosphate dinucleotide bound in the 5′-sensor domain, the central domain with R10 completes the recognition of the 5′-phosphate through a double hydrogen bond interaction (Figure 6A). In the *Sa*RNase W structure, the position of a bound 5′-phosphate (modeled in Figure 6B) would be 4.5 Å away from R10 (R4 in *Sa*RNase W). Instead, R10 makes an H bond with S171 from the 5′-sensor domain.

We hypothesize that the difference in conformation arises from binding and recognition of a 5′-phosphate RNA, which locks the 5′-sensor and S1 domains in a productive conformation through the specific recognition of the 5′-phosphate by R10. This is highly reminiscent of the mechanism of RNase E, where a molecular switch involving an arginine from an opposing monomer is involved in the autoinhibition of the enzyme (49). In RNase W, the conformational modification does not influence the structure of the N-terminal RNase domain, but it changes the relative orientation of the two domains. The regulation of the activity of the RNase domain could therefore arise from the proper presentation of the RNA in the RNase active site by the 5′-sensor and S1 domain (Figure 6C, and Supplementary Video S1).

This hypothesis is largely supported by the fact that the double R10D/R12D mutant has higher RNase activity than the WT protein, indicating that this mutant has lost part of its autoinhibition capacity (Figure 6C). The conserved arginines from the central domain regulate the enzyme’s activity through the binding of a 5′-phosphate containing single-stranded RNA.

In the very conserved patch formed by the central and helical domains, the conserved histidines from the helical domain do not have a clear functional role. RNase activity tests performed with a double alanine mutant of the two conserved histidines H271 and H272 also clearly showed an activation of the RNase activity in the mutant protein, indicating that the histidines, such as arginine R10, are also involved in the regulation of the RNase W-Ct domain RNase activity (Figure 4B and C). Since H271 and H272 are highly conserved among Archaea and H271 interacts with the phosphate ion present in the central domain through a water molecule, we can hypothesize that the histidines participate in the orientation of the RNase W-Ct domain relative to the central domain.

### RNase W facilitates a late step of 16S rRNA maturation in *H. volcanii*

**Figure 7.**
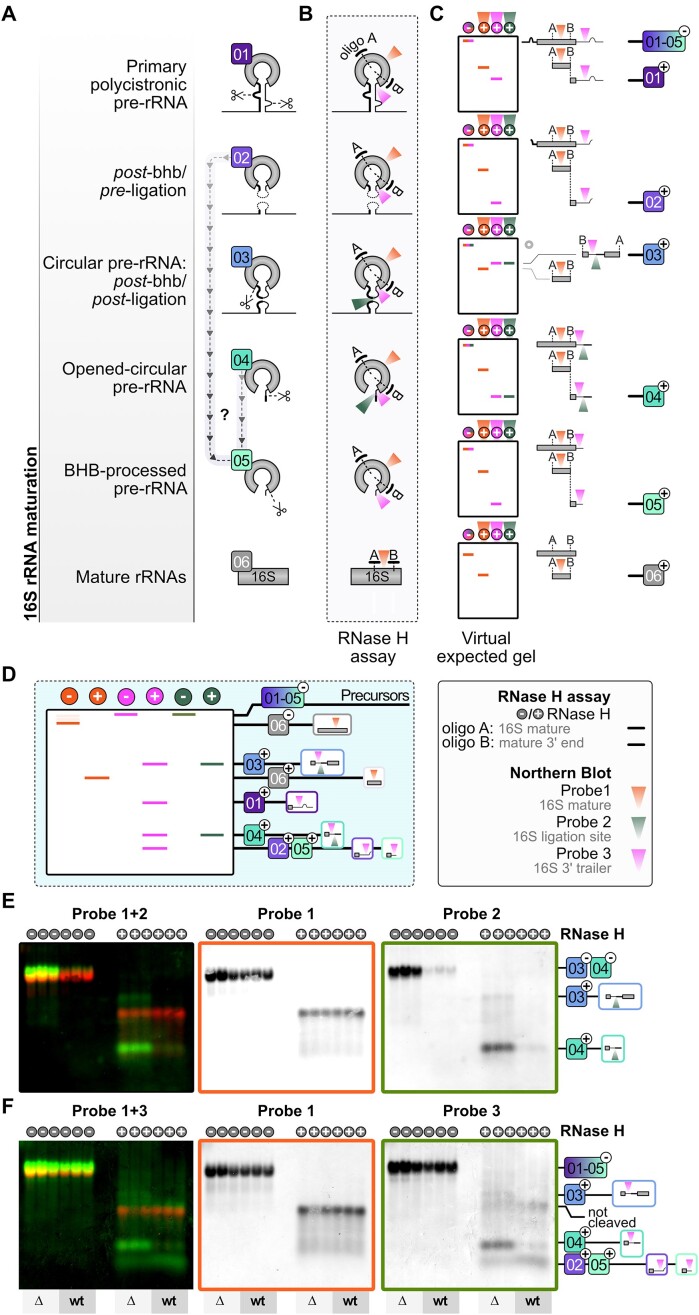
RNase W is required for 16S rRNA maturation in *H. volcanii*. (**A**) Proposed 16S rRNA maturation pathway in *H. volcanii* indicating the different rRNA intermediates, classified as intermediate [1–6] according to Grünberger *et al.* (14). RNA cleavages are indicated by scissors. (**B**) Schematic overview of the RNase H cleavage assay to detect rRNA species [1–6]. Antisense oligos A and B (oHv203 and oHv254, respectively; see the ‘Materials and methods’ section) used for RNase H-dependent cleavage are shown as black lines, while target regions of the respective fluorescently labeled northern blot probes are highlighted by colored triangles. (**C**) Virtual expected gel of each individual maturation stage [1–6] with (+) and without (−) addition of RNase H is depicted. Northern blot probes and expected gel signals after hybridization are color-coded according to the probes used and shown in panel (D). The precursor-specific fragments after RNase H-dependent cleavage (+) are indicated on the right side. (**D**) Summary of the expected signals using total RNA as input for the RNase H cleavage assay. The corresponding precursor-specific fragments and their hybridization characteristic after RNase H-dependent cleavage are indicated (+). Pairwise northern blot analysis using probe 1 (DY782-16S mature region—orange dot)/probe 2 (DY782-16S spacer ligation site—green dot) combination and probe 1 (DY682-16S mature region—orange)/probe 3 (DY782-16S 3′ trailer—pink dot) combination are shown in panels (**E**) and (**F**), respectively. RNAs from WT (H26) and ΔHVO_0406 strains without (−) and with (+) RNase H treatment were analyzed in triplicate. Signals from probes labeled with fluorophore DY682 (probe 1) are shown in red in the merged panel (left panel) and provided as individual panel (middle panel—red box). Signals from probes labeled with fluorophore DY782 (probes 2 and 3) are depicted in green in the merged panel (left panel, E and F, respectively) and provided as individual panels (right panel—green box, E and F, respectively). The precursor-specific fragments after (+) RNase H-dependent cleavage are indicated on the right side. Panels (A)–(D) were adapted from (14) (https://creativecommons.org/licenses/by-nc/4.0/).

To characterize the putative function of RNase W in rRNA maturation, we deleted the gene in the halophilic archaeon *H. volcanii*, a model archaeon that offers several advantages: its genetic manipulation is well established and its rRNA maturation pathway is also one of the best characterized in archaea so far (Figure 7A) (14). We generated the deletion mutant for the *H. volcanii* RNase W open reading frame (HVO_0406) (hereafter called *Hv*RNase W) using the pop-in/pop-out method (47) (see the ‘Material and methods’ section for details). The deletion strain ΔHVO_0406 was successfully generated, indicating that this gene is not essential for cell viability in the laboratory conditions used to generate this strain. Next, we compared the growth behavior of the deletion strain ΔHVO_0406 with the parental WT strain (H119) in various conditions (varying growth temperature and salt concentration in the medium); however, we did not observe any significant growth defect under the conditions analyzed (data not shown).

To investigate the possible role of the *Hv*RNase W in 16S rRNA maturation, we performed northern blot analyses to monitor the presence of pre-rRNA intermediates using fluorescent probes directed against different parts of the flanking spacers, as previously described (14) and as schematically represented in Figure 7 (Figure 7A–D). Since most pre-rRNA intermediates cannot be easily discriminated in size and since the circ-pre-16S rRNA—intermediate [3]—and the opened-circ-pre-16S rRNA—intermediate [4]—have similar hybridization patterns, we have performed RNase H-dependent cleavage using two small DNA oligos hybridizing in the mature region of the 16S rRNA (oligos A and B) as previously established (14) (Figure 7B and C). As indicated, this experimental setup allowed to generate precursor-specific fragments (Figure 7C) that can be discriminated according to their relative migration and hybridization behavior with the indicated probes (Figure 7C and D) (14). In brief, we isolated total RNA from the WT parental strain (H26) and the deletion strain ΔHVO_0406 and subjected them or not to RNase H treatment. All the experiments were performed in triplicates and the resulting RNAs were analyzed by northern blotting using the indicated probes (Figure 7E and F).

In the absence of RNase H treatment, our northern blot analysis revealed an accumulation of 16S pre-rRNAs containing the ligation region of the 5′–3′ spacers generated after circularization of the pre-rRNAs accumulated in the deletion strain (compare probes 2 and 3 in Figure 7E and F). This observation indicates that the *Hv*RNase W is potentially implicated in 16S rRNA maturation, at the level of intermediate [3] and/or [4], the circ-pre-16S rRNA and/or the opened-circ-pre-16S rRNA, respectively (Figure 7A).

As indicated earlier, the main accumulating pre-rRNA species were observed with a probe that cannot distinguish between the two intermediates [3] and [4] (Figure 7A) (14). The opened-circ-pre-16S rRNA—intermediate [4]—differs from the circ-pre-16S rRNA—intermediate [3]—by virtue of its endonucleolytic maturation at the 5′ end of the 16S rRNA by a yet unknown RNase. The resulting opened-circ-pre-16S rRNA—intermediate [4]—is characterized by the presence of a 3′ end extended pre-16S rRNA intermediate containing the ligation of the 3′ end spacer with the most 5′ end of the ETS (External Transcribed Spacer) region (see Figure 7A, and Supplementary Figure S9) (12,14).

To distinguish between these intermediates, we performed RNase H-dependent cleavage to open the circ-pre-16S rRNA molecule [3] or to cleave the opened-circ-pre-16S rRNA intermediates [4] into distinguishable fragments as indicated in Figure 7C (14). The resulting rRNA species were then revealed by northern blot analysis (Figure 7E and F).

This analysis reveals that the opened-circ-pre-16S rRNA intermediates [4] are mostly accumulating in the ΔHVO_0406 strain in comparison with the WT strain, as well as a slight amount of the upstream intermediate, the circ-pre-16S rRNA intermediates [3] (Figure 7E and F). This result suggests that *Hv*RNase W contributes to the processing of pre-rRNA intermediate [4] to intermediate [5] (Figure 7A) (14). Interestingly maturation of pre-rRNA intermediates [4] occurs within a secondary structure generated by the upstream maturation steps leading to the formation of the circ-pre-rRNA intermediates [3]. Moreover, this secondary structure seems common to the three archaea for which we have analyzed the rRNA processing pathway (Supplementary Figure S9) (14). Altogether these results suggest that RNase W might be the RNase responsible for the maturation of pre-16S rRNA intermediate [4] into pre-16S-rRNA intermediate [5] described previously (Figure 7A, and Supplementary Figure S9) (14).

To correlate with our *in vitro* mutagenesis approach, we next performed complementation and rRNA processing analysis with a set of selected *Hv*RNase W mutants (see Supplementary Figure S10 and the ‘Materials and methods’ section). Based on our *in vitro* and structural studies, we have analyzed the effect of point mutations putatively abolishing the RNase activity *in vitro* or N- or C-terminal deletions on 16S rRNA maturation (Supplementary Figure S10). Most of the mutants studied phenocopy the rRNA maturation phenotype observed in the complete deletion strain, suggesting that none of the mutants are able to support the *Hv*RNase W function, apart from *Hv*RNase W H324A. Unfortunately, we were not able to determine the relative expression of all the constructs analyzed and thus this analysis does not allow to fully distinguish between the effects of complete loss of function due to, for example, instability of the respective alleles or the direct effect of the mutations on 16S rRNA maturation. Despite this technical limitation, the complementation analysis further supports the implication of *Hv*RNase W in 16S rRNA maturation.

Collectively, our results provide important new insights into archaeal RNA metabolism and establish RNase W as a new RNase family in Archaea.

## Discussion

### RNase W defines a novel family of RNase involved in 16S rRNA maturation in *H. volcanii*

The evolutionary history of the translation machinery and how the macromolecular adaptation during this history is intertwined with major cellular evolutionary transitions remain to be elucidated in more detail (9–11). The study of ribosome synthesis and function can contribute to a better understanding of cellular evolution and phylogenetic relationships (9–11). However, to do so, ribosome biogenesis and function in archaea remain to be better characterized (9–11).

In this study, we provide functional and structural evidence for a new RNase family, the RNase W family and its involvement in rRNA maturation in archaea. Our structural and functional characterization of the studied archaeal G/E-like–DUF402 hybrid suggests a complex evolutionary history generating a chimeric protein family containing parts of the 5′-sensor and central domains that are characteristic of the RNase G/E-family, which are fused to a domain of unknown function (DUF402), previously annotated as a phosphatase domain. While preparing this manuscript, Kawai *et al.* reported a structure of FAU-1 from *P. furiosus* in agreement with the structures reported in this study (53). In addition, our study demonstrates that the RNase activity is homed within the DUF402 domain and suggests that this activity is inhibited by the presence of the G/E-like domain within the full-length protein. Moreover, Kawai *et al.* reported a homo-trimeric organization, via the C-terminal DUF402 domain, that may have implications for the catalytic regulation of this RNase family (53). However, we did not observe any evidence for this oligomeric state in the conditions we have used. Nevertheless, these observations suggest a complex *in vivo* regulation that might require structural rearrangements to enable timely activation of the catalytic activity. Based on these structural and functional differences and uniqueness in comparison to the bacterial G/E family and to the unique catalytic mechanism of this protein, we proposed to define a new RNase family called RNase W. At this point of our study, it is not yet clear whether isolated DUF402 domains that are found in various bacteria also act as RNases or whether this feature is a characteristic only associated with the G/E-like–DUF402 hybrid encountered in archaea. Further analysis will be necessary to fully characterize the exact function of the bacterial DUF402 domain in regard to RNA metabolism.

Our work provides additional evidence on the existence of late pre-rRNA intermediates previously identified in two independent studies (12,14) and establishes the RNase W protein family as a contributor to the proper maturation of this pre-rRNA intermediate observed in diverse phylogenetically distant archaea (Supplementary Figure S6). Accordingly, our study provides an updated rRNA processing pathway for *H. volcanii* and further positions a processing step mediated by the archaeal-specific RNase W protein in *H. volcanii* and presumably across many other archaeal organisms (Figure 1). Moreover, to our knowledge, our study provides evidence for the first archaeal-specific ribosome biogenesis factor, represented by the RNase W family.

At this stage of our study, it is yet unclear how the substrate of RNase W—the opened-circ-pre-16S rRNA (pre-rRNA intermediate [4]) (14)—is recognized and how cleavage is timely coordinated *in vivo*. Previous functional and structural studies on RNase E demonstrated that the RNase E 5′-sensor domain captures the 5′-phosphate of its substrate, thereby positioning the cleavage within the RNA target at a specific relative distance from the 5′ end of the RNA, in a ruler-like mechanism (29). Moreover, our structural investigation suggests a molecular switch involving structural rearrangement between the 5′-phosphate binding/sensing domain and the catalytic domain that may regulate the catalytic activity. In the cell, this regulation might also involve other protein or RNA co-factor. Based on these data, we proposed that RNase W might interact with the 5′-phosphate of the opened-circ-pre-16S rRNA intermediate via its 5′-phosphate binding domain. We also speculate that this preribosomal intermediate still adopts a circular-like architecture bringing the 5′ and 3′ ends of the 16S rRNA in close proximity, thereby stabilizing these regions in a topologically constraint form facilitating the positioning of the catalytic center of RNase W in the vicinity of its native substrate, the trailer spacer region of the opened-circ-pre-16S rRNA.

Finally, the substrate recognition, catalytic activity regulation and how RNase W activity influences downstream processing events such the final 3′ end maturation of the 16S rRNA presumably performed by archaeal Nob1 (12,13,21) remain to be determined and will be the emphasis of future studies.

## Supplementary Material

gkae907_Supplemental_Files

## Data Availability

Atomic coordinates of the crystal structures reported in this study have been deposited in the Protein Data Bank under accession numbers 8RZA (*Pf*RNase W) and 8RZF (*Sa*RNase W).
