# Supplementary material for: RNase W, a conserved ribonuclease family with a novel active site
Source: Nucleic Acids Res. 2024 Oct 24;52(21):13386–401. doi: 10.1093/nar/gkae907 (PMC11602121; doi:10.1093/nar/gkae907)
Supplement: gkae907_Supplemental_Files [file gkae907_supplemental_files.zip › 24-10-16-SupplementaryData_RNAseW.pdf]

SUPPLEMENTARY MATERIAL TO:

RNase W, a conserved ribonuclease family with a novel active site

Marlène Vayssières[†1], Michael Jüttner[†2], Karina Haas [3], Aurélie Ancelin[1], Anita Marchfelder[3], Nicolas Leulliot*[1], Sébastien Ferreira-Cerca*[2, 4] and Magali Blaud*[1]

[1] Université Paris Cité, CNRS, CiTCoM, F-75006, Paris, France

[2] Regensburg Centre for Biochemistry, Biochemistry III – Institute for Biochemistry, Genetics and Microbiology, University of Regensburg, Universitätsstraße 31, 93053 Regensburg, Germany.

[3] Molecular Biology and Biotechnology of Prokaryotes, Ulm University, 89069 Ulm, Germany

[4] Laboratoire de Biologie Structurale de la Cellule (BIOC), UMR 7654—CNRS, École polytechnique, Institut Polytechnique de Paris, Route de Saclay, Palaiseau 91128, France.

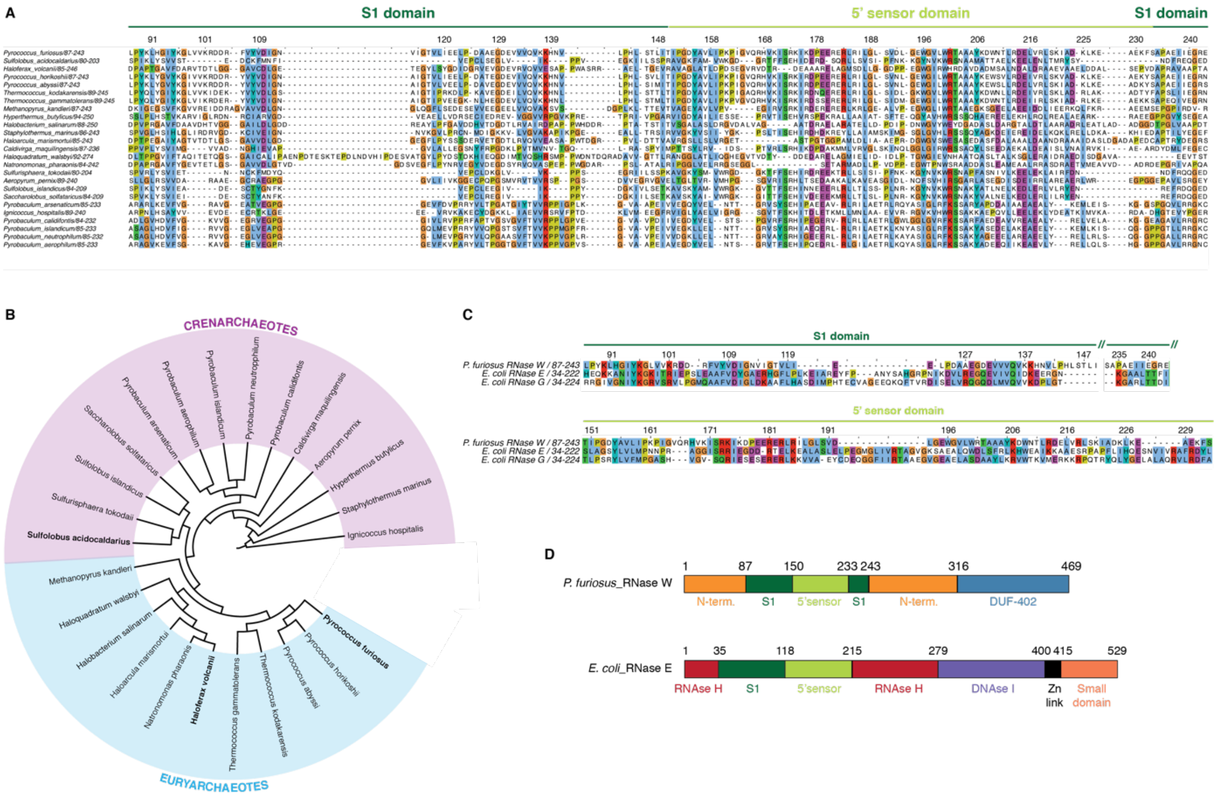

Figure S1. Multiple sequences alignments (MSA) of RNA recognition domain. (**A**) Clustal Omega local alignments of S1 and 5'-sensor domains from 27 archaeal homologs obtained with BlastP research against UniprotKB/Swiss-Prot data bank (1–3). MSA were visualized in Jalview (4) and colored based according to the biochemical properties of amino acids. (**B**) Phylogenetic tree obtained by neighbor-joining from Clustal Omega-MSA shown in A. G/E-like domain composed of S1 and 5'-sensor subdomains are present in crenarchaeotes and euryarchaeotes. (**C**) Clustal Omega local alignments of S1 and 5'sensor domains from *P. furiosus* RNase W, *E. coli* RNase E, and RNase G. MSA were visualized in Jalview (4) and colored based according to the biochemical properties of amino acids. (**D**) Schematic representation of domain organization of *P. furiosus* RNase W and *E. coli* RNase E. The domain organization of *E. coli* RNase G is similar to its paralog RNase E. RNase H and DNase I are the catalytic domain of RNase E but these domains are absent from *Pf*RNase W. Only the RNA recognition domain (S1 / 5'-sensor subdomains) is present in the two proteins.

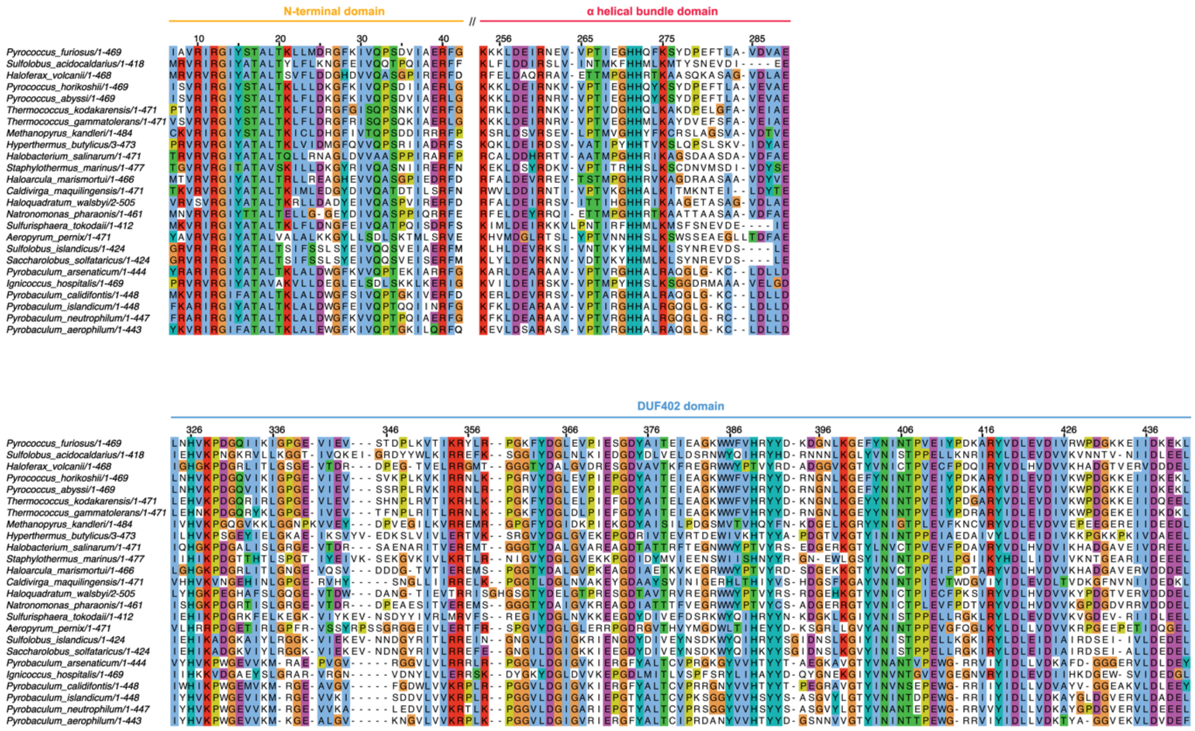

Figure S2. Multiple sequence alignments (MSA) on conserved regions of *Pf*RNase W.

Firstly, a BlastP research against UniprotKB/Swiss-Prot database (1, 2) using *Pf*RNase W as a query was done to obtain a list of homolog proteins. Then, local MSA on the homolog sequences were done separately on N-terminal domain, α helical bundle domain and DUF402 domain using clustal W (3). Amino acids are colored according to their physiochemical properties.

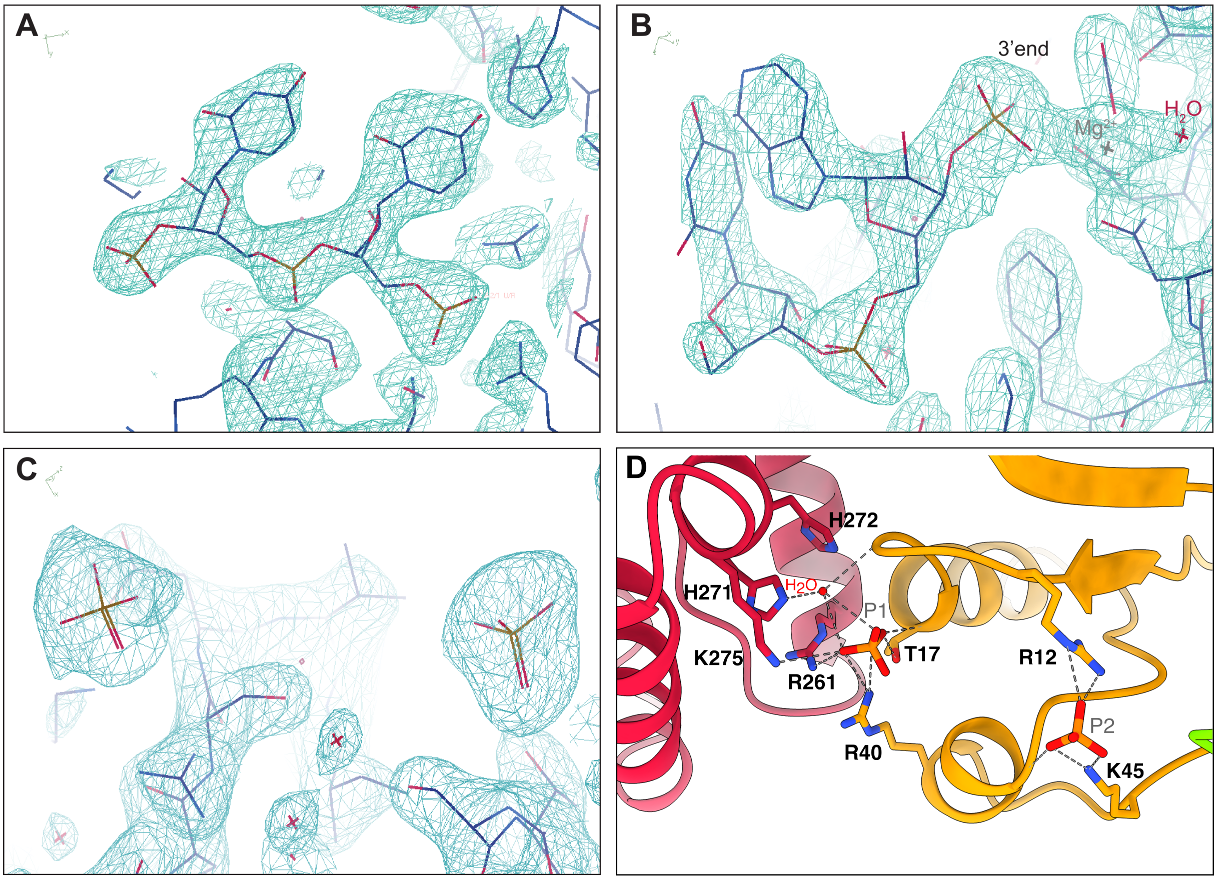

Figure S3. Density maps of *Pf*RNase W in ligands regions. (**A**) Dinucleotide UU in dark blue refined in density map *Pf*RNase W in cyan. (**B**) Dinucleotide UA in dark blue refined in density map *Pf*RNase W in cyan. (**C**) Two phosphates model in density map *Pf*RNase W in cyan. **(D)** The two phosphates bound in the central domain are annotated P1 and P2.

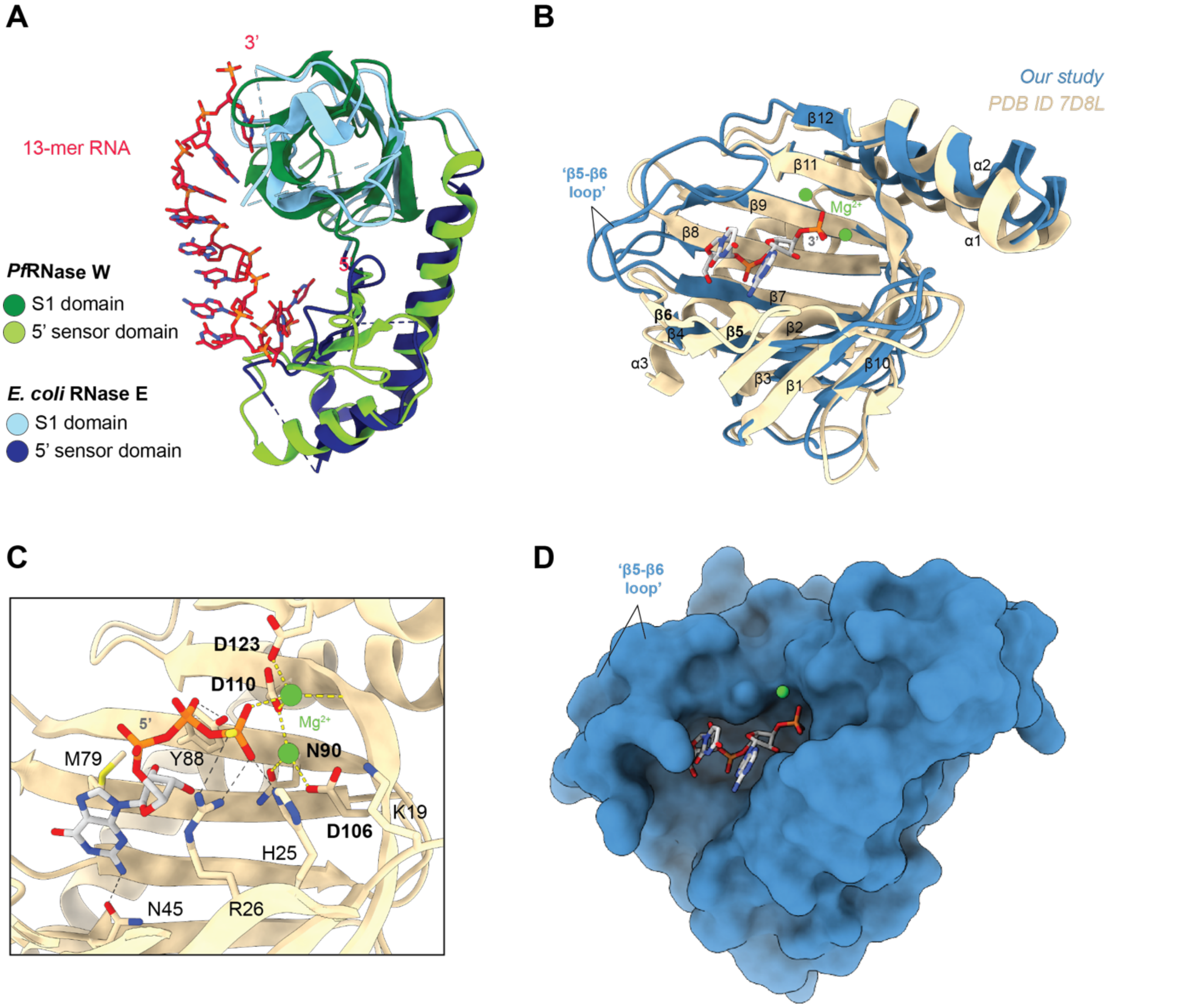

Figure S4. Comparison of structural homologs with domains of *Pf*RNase W. (**A**) Superposition of S1 and 5'-sensor domains of *Pf*RNase W and *E. coli* RNase E in complex with a 13-mer RNA (PDB ID 2C0B) (5). For clarity, only S1 and 5'-sensor domains are shown for both structures. The S1 and 5'-sensor domains of *Pf*RNase W form an interaction surface for the binding of a 13-mer single-stranded RNA as in the structure of RNase E. **(B)** Superposition of structural homolog Ntdp nucleoside tri- and di-phosphatase from *Staphylococcus aureus* (in beige, PDB ID 7D8L) (6) to *Pf*RNase W (in blue). A dinucleotide UA with a 3'phosphate end (in light grey) is bound in the central cavity of the *Pf*RNase W DUF402 domain as well as two magnesium ions. **(C)** GTP-γS is bound in the Ntdp nucleoside phosphatase from *Staphylococcus aureus* (in beige, PDB ID 7D8L) (6). The protein is in the same orientation as in **Fig. S2**. Interactions between the ligand, magnesium ions, and amino acids are illustrated with dashed lines. In bold, the equivalent residues present in the *Pf*RNase W putative phosphatase domain are indicated. (**D**) Structure of DUF402 domain of *Pf*RNase W, in the same orientation as in (B): the β5-β6 loop creates a narrow groove which can accommodate several nucleotides at the 5' and 3' end.

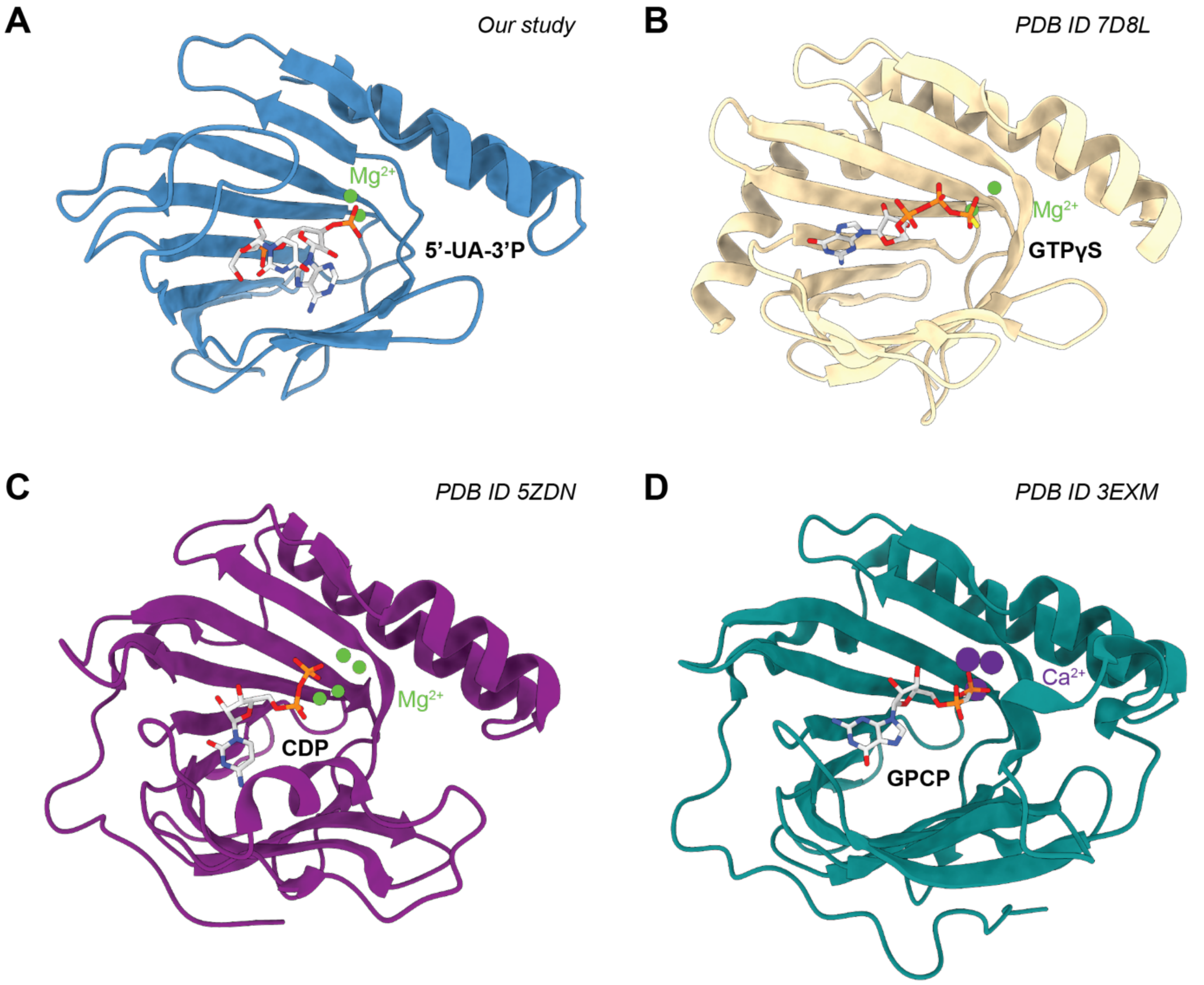

Figure S5. DUF402 domain phosphatase family. (**A**) Structure of the putative phosphatase domain of *Pf*RNase W. (**B**) Structure of Ntdp nucleoside tri- and di-phosphatase from *Staphylococcus aureus* involved in bacterial virulence (6). (**C**) Structure of FomD Cytidylyl (S)-2-Hydroxypropylphosphonate hydrolase involved in Fosfomycin Biosynthesis (7). (**D**) Structure of SC4828 phosphatase from *Streptomyces coelicolor*.

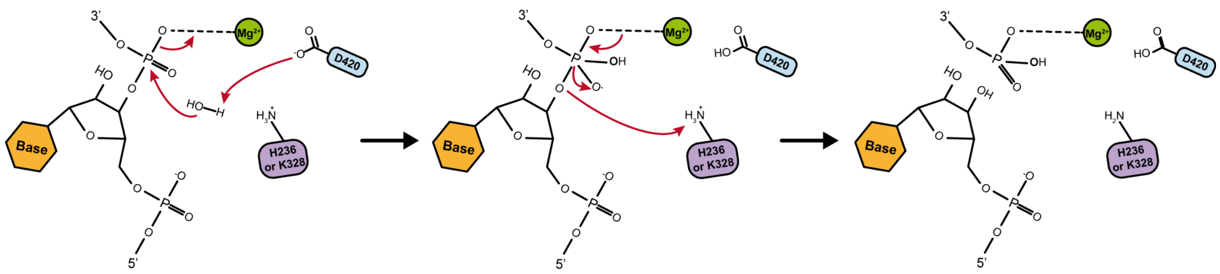

Figure S6. Proposed catalytic reaction to cleave the RNA substrate, adapted from (6)**.**

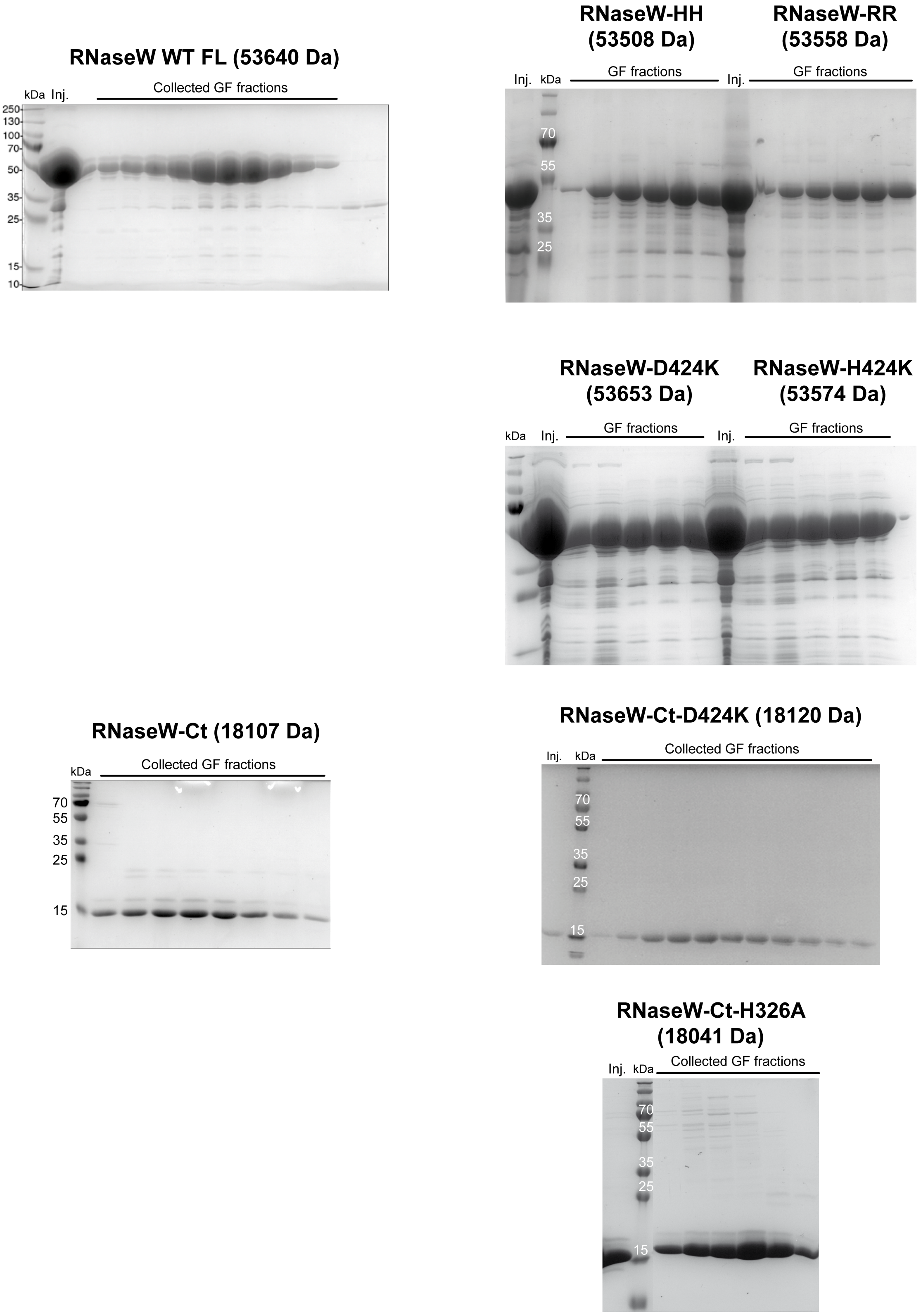

Figure S7. SDS-PAGE analysis of all the RNase W wild type and mutant recombinant proteins used in the ribonuclease assays. The gels correspond to the analysis of elution fractions of the gel filtration. Inj : sample injected in the gel filtration column. Name and molecular weight of the wild-type and mutant proteins are indicated above each SDS-PAGE.

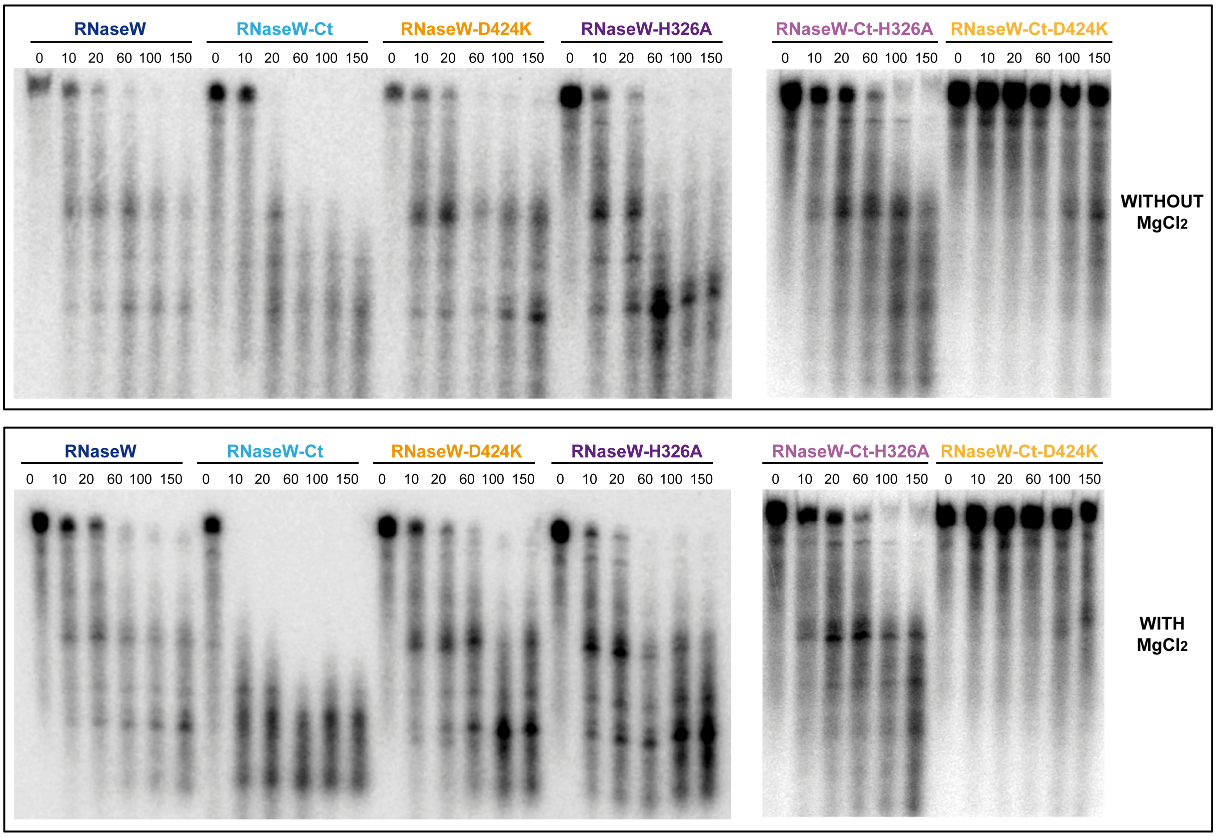

Figure S8. Influence of magnesium on the RNase activity of WT, C-terminal domain of *Pf*RNase W and mutant proteins. A concentration of 0.5 mM of $MgCl_2$ was used in the assay shown in the bottom panel (with $MgCl_2$). Similar results are observed for each protein with or without magnesium in the reaction mixture.

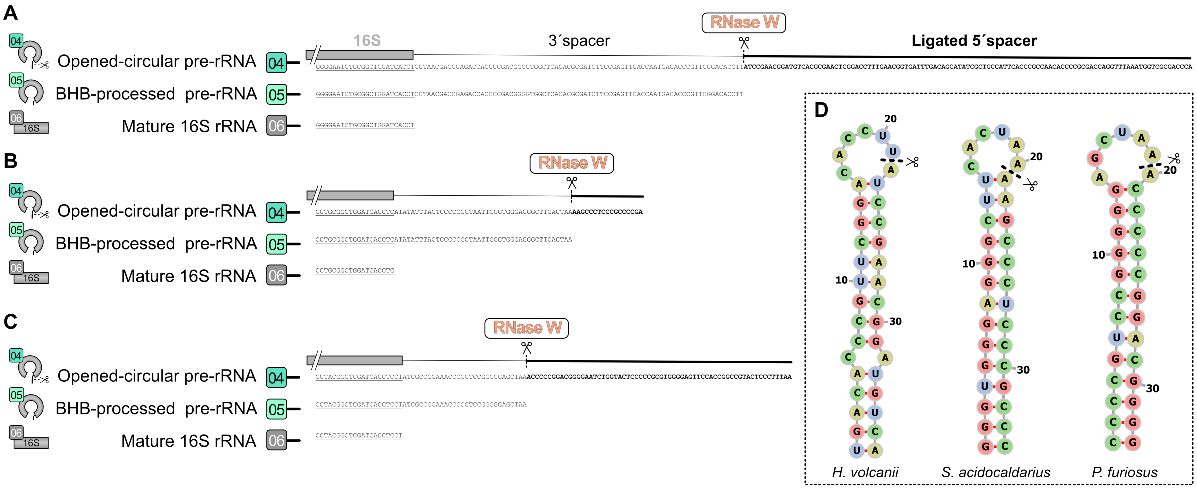

Figure S9. Sequence and structural features of the processing site of pre-rRNA intermediates [4] generating intermediates [5]

Sequences of the 3´end of the (pre-)rRNA intermediates [4-6] as previously determined by Nanopore sequencing for *H. volcanii* (A), *S. acidocaldarius* (B), and *P. furiosus* (C) are depicted (8). The cleavage sites in the pre-rRNA intermediates [5] presumably carried by RNAse W are indicated by scissors. (D) Secondary structure predictions (9) of the rRNA region flanking the putative RNase W cleavage sites (scissors) in three archaea are provided.

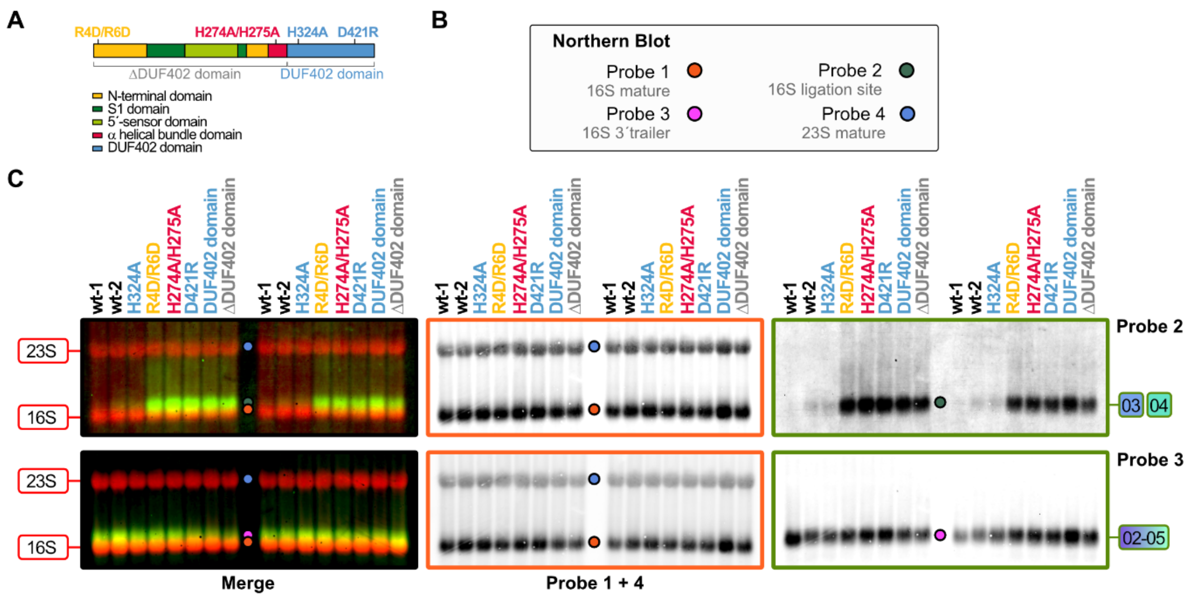

Figure S10. ***In vivo*** **complementation analysis of RNase W deletion strain in** ***H. volcanii***
Total RNA from markerless deletion strain ΔHVO_0406 complemented with pTA942-*Hv*RNase W, wild-type protein (fused to an N-terminal or C-terminal 3x Flag-tag, wt-1 and wt-2, respectively); and the indicated *Hv*RNase W mutants: R4A/R6D, H274D/H275D, H324A, D421R, C-terminal putative phosphatase domain only, N-terminal-domain (lacking the putative phosphatase domain), all fused to a C-terminal 3x Flag-tag, were analyzed by Northern blot analysis. **(A)** Domain organization of *Hv*RNase W, and positioning of the respective mutations used in this study. **(B)** Probes used for Northern blot analysis shown in panel C are indicated. **(C)** Single and merged signals from Northern blot analysis performed with probes 1, 2, 3, and 4 and the corresponding rRNA intermediates [1-6] are indicated according to Figure 7 and (8). Triple hybridization using probe 1 (DY782-16S mature region – orange dot)/ probe 2 (DY782-16S spacer ligation site – green dot)/ probe 4 (DY682-23S mature region – blue dot) combination and triple hybridization using probe 1 (DY682-16S mature region - orange)/ probe 3 (DY782-16S 3´trailer – pink dot)/ probe 4 (DY682-23S mature region – blue dot) combination are shown in upper and lower panel, respectively. Signals from probes labeled with fluorophore DY682 (probes 1 and 4) are shown in red in the merged panel (left panels) and provided as individual panels (middle panels - red box). Signals from probes labeled with fluorophore DY782 (probes 2 and 3) are depicted in green in the merged panels (left panels) and provided as individual panels. The nature of the mature and pre-rRNA based on hybridization on respective hybridization behaviors are indicated on the left and right sides, respectively. The analyzed mutants are color-coded according to RNase W domain positioning of the respective mutations as depicted in (A).

VIDEOS

Supplementary Video 1

The 13-mer RNA from the structure of the *E. coli* RNAse E (PDB ID 2C0B) was used to model the binding of RNA in the *Sa*RNAse W and *Pf*RNAse W structures. To do so, S1 and 5'-sensor domains of the RNAse E in complex with this RNA and *Sa*RNAse W were superimposed. The same procedure was done with *Pf*RNAse W. Modelled structures of *Sa*Rnase W and *Pf*RNAse W with 13-mer RNA were superimposed on their N-terminal domain. Then, a morph conformation between the two modelled structures was done and recorded to make this video of the proposed conformational changes of *Pf*RNAse W upon RNA binding.

This video shows our hypothesis of the movements of the different domains of the *Pf*RNAse W upon RNA binding: S1 (dark green) and 5'-sensor domains (light green) are rotated downwards while α-helical bundle domain (red) and DUF402 domain (blue) are rotated upwards. These tilting result in the fitting of the RNA in the protein, especially in the DUF402 domain where the ribonuclease activity takes place.

Supplementary References

1. Duvaud,S., Gabella,C., Lisacek,F., Stockinger,H., Ioannidis,V. and Durinx,C. (2021) Expasy, the Swiss Bioinformatics Resource Portal, as designed by its users. *Nucleic Acids Research*, **49**, W216–W227.

2. The UniProt Consortium (2023) UniProt: the Universal Protein Knowledgebase in 2023. *Nucleic Acids Research*, **51**, D523–D531.

3. Thompson,J.D., Higgins,D.G. and Gibson,T.J. (1994) CLUSTAL W: improving the sensitivity of progressive multiple sequence alignment through sequence weighting, position-specific gap penalties and weight matrix choice. *Nucleic Acids Res.*, **22**, 4673–4680.

4. Waterhouse,A.M., Procter,J.B., Martin,D.M.A., Clamp,M. and Barton,G.J. (2009) Jalview Version 2—a multiple sequence alignment editor and analysis workbench. *Bioinformatics*, **25**, 1189–1191.

5. Callaghan,A.J., Marcaida,M.J., Stead,J.A., McDowall,K.J., Scott,W.G. and Luisi,B.F. (2005) Structure of Escherichia coli RNase E catalytic domain and implications for RNA turnover. *Nature*, **437**, 1187.

6. Wang,Z., Shen,H., He,B., Teng,M., Guo,Q. and Li,X. (2021) The structural mechanism for the nucleoside tri- and diphosphate hydrolysis activity of Ntdp from Staphylococcus aureus. *The FEBS Journal*, **288**, 6019–6034.

7. Sato,S., Miyanaga,A., Kim,S.-Y., Kuzuyama,T., Kudo,F. and Eguchi,T. (2018) Biochemical and Structural Analysis of FomD That Catalyzes the Hydrolysis of Cytidylyl (S)-2-Hydroxypropylphosphonate in Fosfomycin Biosynthesis. *Biochemistry*, **57**, 4858–4866.

8. Grünberger,F., Jüttner,M., Knüppel,R., Ferreira-Cerca,S. and Grohmann,D. (2023) Nanopore-based RNA sequencing deciphers the formation, processing, and modification steps of rRNA intermediates in archaea. *RNA*, **29**, 1255–1273.

9. Gruber,A.R., Lorenz,R., Bernhart,S.H., Neuböck,R. and Hofacker,I.L. (2008) The Vienna RNA Websuite. *Nucleic Acids Research*, **36**, W70–W74.
